# GLUT1 is redundant in hypoxic and glycolytic nucleus pulposus cells of the intervertebral disc

**DOI:** 10.1172/jci.insight.164883

**Published:** 2023-04-24

**Authors:** Shira N. Johnston, Elizabeth S. Silagi, Vedavathi Madhu, Duc H. Nguyen, Irving M. Shapiro, Makarand V. Risbud

**Affiliations:** 1 Department of Orthopaedic Surgery, Sidney Kimmel Medical College, and; 2 Graduate Program in Cell Biology and Regenerative Medicine, College of Life Sciences, Thomas Jefferson University, Philadelphia, Pennsylvania, USA.

**Keywords:** Bone Biology, Metabolism, Cartilage, Glucose metabolism, Hypoxia

## Abstract

Glycolysis is central to homeostasis of nucleus pulposus (NP) cells in the avascular intervertebral disc. Since the glucose transporter, GLUT1, is a highly enriched phenotypic marker of NP cells, we hypothesized that it is vital for the development and postnatal maintenance of the disc. Surprisingly, primary NP cells treated with 2 well-characterized GLUT1 inhibitors maintained normal rates of glycolysis and ATP production, indicating intrinsic compensatory mechanisms. We showed in vitro that NP cells mitigated GLUT1 loss by rewiring glucose import through GLUT3. Of note, we demonstrated that substrates, such as glutamine and palmitate, did not compensate for glucose restriction resulting from dual inhibition of GLUT1/3, and inhibition compromised long-term cell viability. To investigate the redundancy of GLUT1 function in NP, we generated 2 NP-specific knockout mice: *Krt19^CreERT^ Glut1^fl/fl^* and *Foxa2^Cre^ Glut1^fl/fl^*. There were no apparent defects in postnatal disc health or development and maturation in mutant mice. Microarray analysis verified that GLUT1 loss did not cause transcriptomic alterations in the NP, supporting that cells are refractory to GLUT1 loss. These observations provide the first evidence to our knowledge of functional redundancy in GLUT transporters in the physiologically hypoxic intervertebral disc and underscore the importance of glucose as the indispensable substrate for NP cells.

## Introduction

The phenotype of nucleus pulposus (NP) cells reflects their notochordal origin and unique hypoxic environment (1–3). Indeed, an initial definition of the NP cell phenotype was based on the observation that the HIF1α transcription factor and glucose transporter, GLUT1, were highly enriched in the NP and not the annulus fibrosus (AF) compartment of the intervertebral disc (4). To facilitate hypoxic adaptation, NP cells constitutively express HIF1α, given that the vasculature in the adjacent vertebral bodies does not penetrate the NP compartment (5, 6). HIF1α promotes the biosynthetic activity of NP cells by regulating glycolytic metabolism and mitochondrial TCA cycle flux, and conditional deletion of *Hif1a* in notochord results in massive NP cell apoptosis at birth probably due to metabolic failure (7–9). Further investigations into the HIF1α transcriptional program showed that Glut1 was the HIF1α target, and expression was regulated through prolyl hydroxylase 3–dependent modulation of HIF1α–C-terminal transactivation domain activity (10).

It has been well established that the maintenance of glycolytic flux and nutrient-metabolite balance is critical for cell survival in the intervertebral disc. Glucose passively diffuses from the vertebral capillaries, through the hyaline cartilaginous endplates and proteoglycan-rich extracellular matrix of the NP tissue compartment, to reach the resident NP cells at the center of the disc. Despite this long diffusion distance, glucose levels must surpass a critical threshold for cells to remain viable; glucose concentrations below 0.5 mM are shown to promote cell death (11). Numerous studies have confirmed that glucose availability is required for critical NP cellular processes such as protein and proteoglycan biosynthesis, glycolytic flux, and maintaining cell viability (12–15). Furthermore, disruption in the balance between glucose consumption and lactic acid production can significantly affect NP cell physiology (16, 17). Several factors can influence the rate of glucose consumption in NP cells, including nutrient deprivation from decreased glucose diffusivity or increased cell density (18), pH buffering capacity governed by relative levels of bicarbonate and sodium (19), mechanical stress (20), and oxygen tension (21, 22).

Despite the well-studied importance of glucose availability and consumption on NP cell viability in vitro, few studies have focused on the relationship between glucose consumption and disc health in vivo. This is due to the complexity of studying solute transport and metabolite concentrations in animal models or by using genetic techniques. However, some studies have shown enriched expression of GLUT1 in human NP and that GLUT3 and GLUT9 levels are lower than GLUT1 (23). These results suggest that glucose transporter redundancy may be required for disc health and function.

Considering that GLUT1 is a high-affinity glucose transporter with nearly ubiquitous expression in all tissue types, it is not surprising that embryos with homozygous *Glut1* deficiency are nonviable (24). *Glut1* haploinsufficiency in mice causes profound developmental defects recapitulating those seen in human patients with GLUT1 deficiency syndrome (25). Both humans and mice with *Glut1* haploinsufficiency experience microcephaly, impaired motor function, epileptiform changes on EEG, and hypoglycorrhachia (25). Due to the severe impact of loss of GLUT1 on gross embryonic development, tissue-specific knockout mice have been used to delineate the role of GLUT1 in developing skeleton and other connective tissues (26–30). These studies show that *Glut1* deletion leads to metabolic reprogramming of cells, profound phenotypic changes, and compromised tissue function. In skeletal tissues, loss of *Glut1* expression results in severely impaired bone development, emphasizing the importance of GLUT1 in the maintenance of bone health. As far as mechanisms are concerned, it is known that GLUT1 expression precedes transcription factor *Runx2* and is required for promoting bone formation by blocking AMPK-dependent degradation of RUNX2 (27). Mouse models of *Glut1* loss of function in the growth plate and articular cartilage demonstrate that *Glut1* function is required for cartilage structure and function regulating cell proliferation, matrix production, and resistance to injury and osteoarthritis (31). GLUT1 expression is controlled by a unique BMP/mTORC1/HIF1 signaling cascade in chondrocytes where it is required for chondrocyte proliferation and hypertrophy (28).

Since GLUT1-mediated glycolytic metabolism plays a fundamental role in many tissues, including bone and cartilage homeostasis, we surmised that loss of GLUT1 expression in the NP would affect both development and age-related structure of the disc (26–30). However, unlike the other skeletal tissues, both conditional and inducible loss of GLUT1 expression in the NP did not result in notable degenerative changes in the discs of developing perinatal mice or in skeletally mature mice. Surprisingly, microarray analysis of global transcriptomic changes in NP tissue isolated from conditional *Glut1*-knockout mice did not reveal any differentially regulated genes besides *Slc2a1/Glut1* — a finding suggesting NP cells are refractory to loss of GLUT1. In fact, long-term GLUT1 inhibition had no effect on the rates of NP glycolytic flux or oxidative metabolism; these findings indicate that NP cells can potentially mitigate the loss of GLUT1 function by rewiring glucose import through GLUT3. Importantly, our findings suggest that under glucose-limiting conditions resulting from dual inhibition of GLUT1/3, NP cells do not evidence metabolic reprogramming to utilize alternative substrates, such as glutamine and fatty acids. These findings provide the first evidence to our knowledge of functional redundancy in GLUT transporters in a physiologically hypoxic intervertebral disc and underscore the importance of glucose as the indispensable metabolic substrate for NP cells.

## Results

### HIF1-dependent GLUT1 expression is highly enriched in the NP and declines with age.

**Figure 1:**
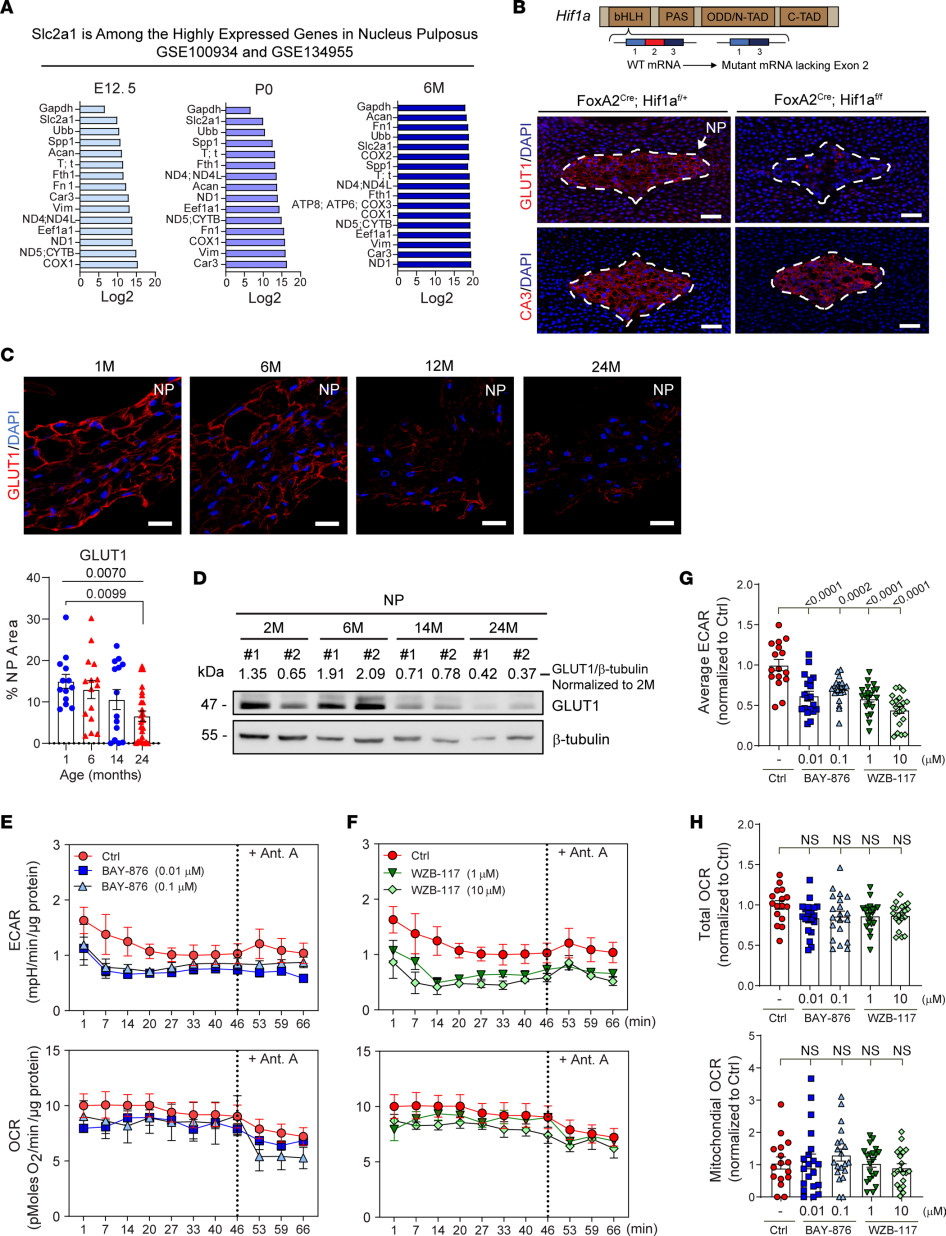
GLUT1, an enriched NP marker, is an HIF1α target and its expression declines with aging. (**A**) Graph showing select highly expressed genes in mouse NP cells at E12.5, P0 (Gene Expression Omnibus GSE100934) (32), and 6 months (Gene Expression Omnibus GSE134955) (33). *Glut1* is among the top 2.5% expressed transcripts at each of the time points. (**B**) Schematic showing deletion of *Hif1a* exon 2 to generate mutant *Hif1a* mRNA. Representative IHC images of NP cell phenotypic markers GLUT1 and CA3 in *Hif1a* WT and *Hif1a*^cKO^ (*Foxa2^Cre^ Hif1a^fl/fl^*) mice (scale bar = 50 μm). (**C**) Representative images and quantification of GLUT1 in C57BL/6J mice with aging (scale bar = 25 μm) (*n* = 5 mice/time point; 3–6 discs/animal; 15–30 discs/time point). (**D**) Western blot showing GLUT1 levels in mouse NP with aging (*n* = 2 mice/time point; 20 discs/animal were pooled); the blot was first probed for GLUT1, stripped, and reprobed for β-tubulin. (**E**) Effect of short-term (1-hour) GLUT1 inhibition by BAY-876 on ECAR and OCR measurements in NP cells. (**F**) Effect of short-term GLUT1 inhibition by WZB-117 on ECAR and OCR measurements in NP cells. (**G**) Short-term GLUT1 inhibition by BAY-876 and WZB-117 results in decreased average ECAR. (**H**) Short-term GLUT1 inhibition does not affect total OCR in BAY-876– and WZB-117–treated cells. Mitochondrial OCR measurements following short-term treatment by BAY-876 and WZB-117. (*n* = 6 independent experiments, 4 technical replicates/experiment/group.) Quantitative measurements represent mean ± SEM. Significance was determined using a 1-way ANOVA (**G** and **H**) or Kruskal-Wallis (**C**) with Dunnett’s or Dunn’s post hoc test as appropriate.

During embryogenesis, the notochord and developing NP compartment of the intervertebral disc are hypoxic and exhibit robust HIF1α activity. Our previous work has shown that notochord-specific *Hif1a* deletion in *Foxa2^Cre^* *Hif1a^fl/fl^* (*Hif1a*^cKO^) mice leads to massive NP cell death at birth, probably due to metabolic failure of cells that rely primarily on glycolytic metabolism for their energetic needs (7–9). It is therefore not surprising that through early development to skeletal maturity, expression of the glucose transporter, *Slc2a1/Glut1*, was highly enriched in NP cells, was among the top 2.5% expressed transcripts (Figure 1A), and is regarded as a phenotypic marker (3, 32, 33). In fact, GLUT1 expression is substantially decreased in the NP of E15.5 *Hif1a*^cKO^ mice without affecting the level of carbonic anhydrase 3 (CA3), another hypoxia-sensitive NP phenotypic marker (34) (Figure 1B). These results indicate that loss of GLUT1 and consequent restriction on glucose availability shortly precedes the catastrophic NP cell death observed in *Hif1a*^cKO^ mice at birth. Furthermore, changes in GLUT1 expression in the intervertebral disc correlate to both age and degenerative state (23). Our studies show that in mice, GLUT1 expression is significantly decreased during normal aging from 1 month to 24 months (Figure 1, C and D). Immunofluorescence staining of GLUT1 shows a robust expression of GLUT1 in the NP compartment at early time points, followed by a significant decrease in GLUT1 abundance by 24 months of age (Figure 1C). In fact, Western blot verified that GLUT1 protein level was markedly decreased by as early as 14 months of age (Figure 1D). Based on these findings, we hypothesized that GLUT1 is critically important for NP cell survival and function.

### Long-term inhibition of GLUT1 does not affect glycolytic or oxidative metabolism in NP cells.

To determine if the loss of GLUT1 function directly impairs NP cell metabolism, we modeled the loss of GLUT1 in NP cells in vitro with 2 highly specific pharmacological inhibitors; namely, BAY-876 and WZB-117 (35, 36). Using a Seahorse Flux Analyzer, we assessed metrics of NP cell glycolytic flux by measuring extracellular acidification rate (ECAR) and oxidative flux by oxygen consumption rate (OCR). After short-term inhibition of GLUT1 for 1 hour, NP cells showed significantly decreased average ECAR with both inhibitors (Figure 1, E–G). However, total OCR and mitochondrial OCR remained unchanged, suggesting the NP cells did not undergo a metabolic switch from glycolytic to oxidative metabolism (Figure 1, E, F, and H). Moreover, inhibition of the electron transport chain (ETC) by antimycin A failed to influence mitochondrial respiration. It is concluded that while the baseline mitochondrial respiration remains low in both control and inhibitor-treated cells, this is not related to failure to provide metabolic intermediates to power the ETC (Figure 1, E and F).

**Figure 2:**
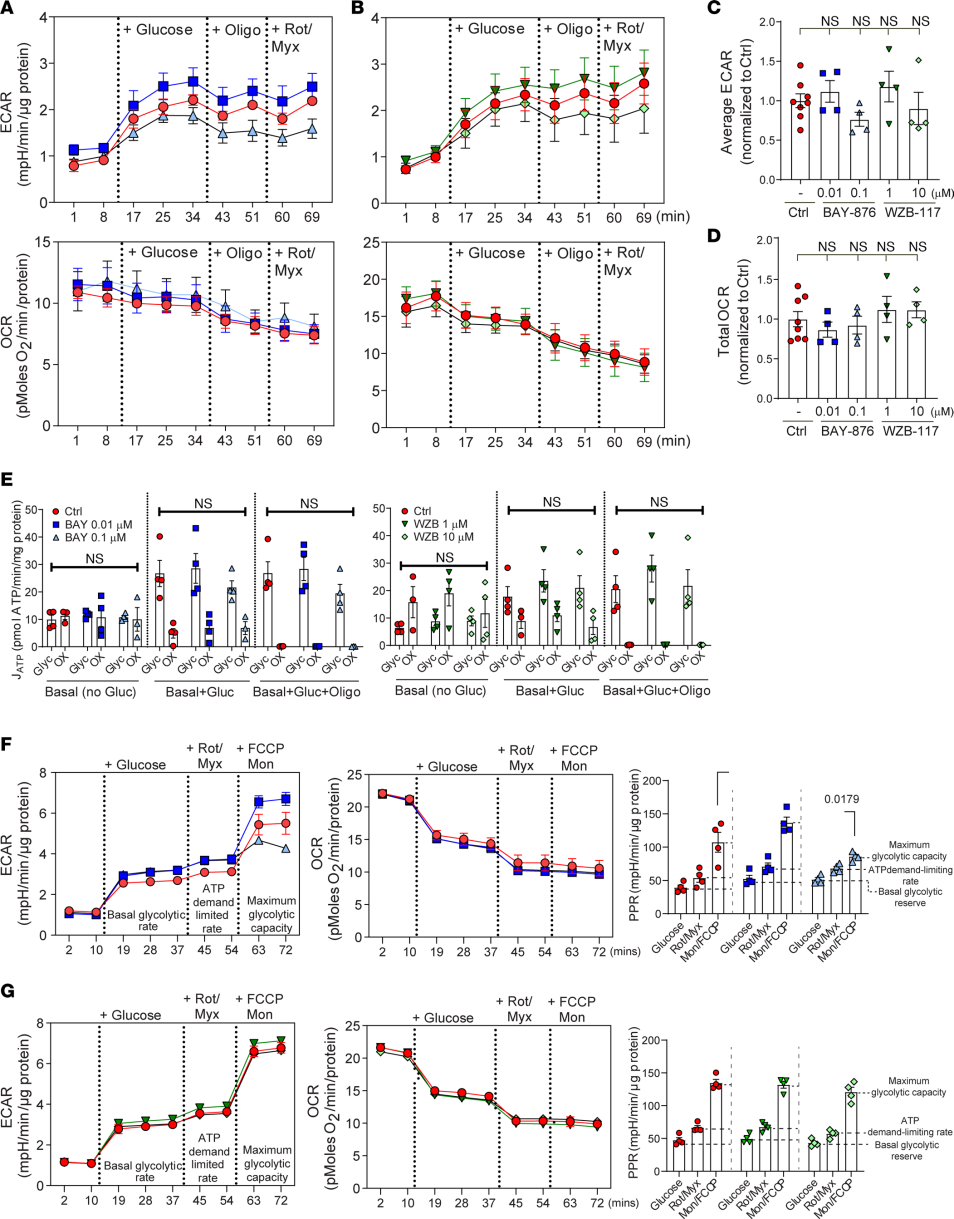
Long-term inhibition of GLUT1 does not affect glycolytic or oxidative metabolism in NP cells. (**A**) ECAR and OCR profiles following long-term GLUT1 inhibition (24 hours) by BAY-876 in NP cells. (**B**) ECAR and OCR profiles following long-term GLUT1 inhibition (24 hours) by WZB-117. (**C** and **D**) Long-term GLUT1 inhibition by BAY-876 and WZB-117 did not alter (**C**) ECAR and (**D**) OCR measurements. (**E**) Long-term GLUT1 inhibition by BAY-876 and WZB-117 did not affect ATP production by NP cells. Gly, glycolytic ATP production; OX, oxidative ATP production. (**F**) ECAR and OCR traces in 24-hour BAY-876–treated and control NP cells to assess glycolytic capacity and reserve. inhibition of GLUT1 by BAY-876 minimally affects glycolytic reserve by NP cells only at the higher concentration of 0.1 μM. (**G**) ECAR and OCR traces in 24-hour WZB-117–treated and control NP cells to assess proton production rate. Inhibition of GLUT1 by WZB-117 does not alter the proton production rate by NP cells. Quantitative measurements represent mean ± SEM (*n* = 4 biological replicates, 4 technical replicates/experiment/group). Significance was determined using 1-way ANOVA.

To determine if longer term inhibition of GLUT1 influences NP cell metabolism, we measured ECAR and OCR and calculated the ATP production rates from glycolysis and oxidative metabolism in NP cells treated with 2 concentrations of BAY-876 and WZB-117 for 24 hours. We recorded raw ECAR and OCR traces under basal conditions (no glucose), followed by sequential addition of glucose (substrate), oligomycin (ATP synthase inhibitor), and rotenone + myxothiozol (ETC inhibitors) (Figure 2, A and B). Surprisingly, there were no significant differences in the average ECAR and OCR between control and GLUT1-inhibited NP cells (Figure 2, C and D), as was also evident from the raw tracer profiles. Using the published method by Mookerjee and colleagues (37), we calculated glycolytic and oxidative ATP production rates following GLUT1 inhibition with BAY-876 and WZB-117. We estimated that under basal conditions (no glucose), oxidative metabolism generated ~50% of ATP in control NP cells; however, this decreased to ~10%–25% when glucose was added (Figure 2E). Importantly, our results showed that there was no difference in ATP production rates from glycolysis or oxidative phosphorylation in GLUT1-inhibited cells as compared to controls (Figure 2E). Furthermore, blocking oxidative ATP production entirely with oligomycin showed strikingly little effect on glycolytic ATP production rate, suggesting that glycolytic flux is largely independent of oxidative metabolism in NP cells (Figure 2E). To understand if there was an effect of GLUT1 loss on glycolytic reserve, we measured ECAR and OCR and calculated the proton production rate (PPR) in NP cells treated with 2 concentrations of BAY-876 and WZB-117 for 24 hours. We recorded ECAR and OCR traces under basal conditions (no glucose), followed by sequential addition of glucose, rotenone (Rot) + myxothiozol (Myx) (ETC inhibitors), and carbonyl cyanide *p*-(tri-fluromethoxy)phenyl-hydrazone (FCCP), an uncoupler of mitochondrial oxidative phosphorylation, + monensin (Mon), which decreases the mitochondrial membrane potential (Figure 2, F and G). There were minimal differences in the average ECAR and OCR between control and GLUT1-inhibited NP cells (Figure 2, F and G) and accordingly a minimal difference in the PPR and glycolytic reserve of GLUT1-inhibited cells as compared to controls (Figure 2, F and G). Taken together, the results of the Seahorse metabolic assays suggested that inhibiting glucose uptake through GLUT1 in NP cells caused an immediate decrease in glycolytic flux. However, compensatory mechanisms are capable of restoring NP cell glycolytic metabolism within 24 hours, without initiating a metabolic shift toward oxidative metabolism or affecting the glycolytic capacity and reserve of the cells.

### GLUT3 sustains glucose uptake in the absence of GLUT1.

**Figure 3:**
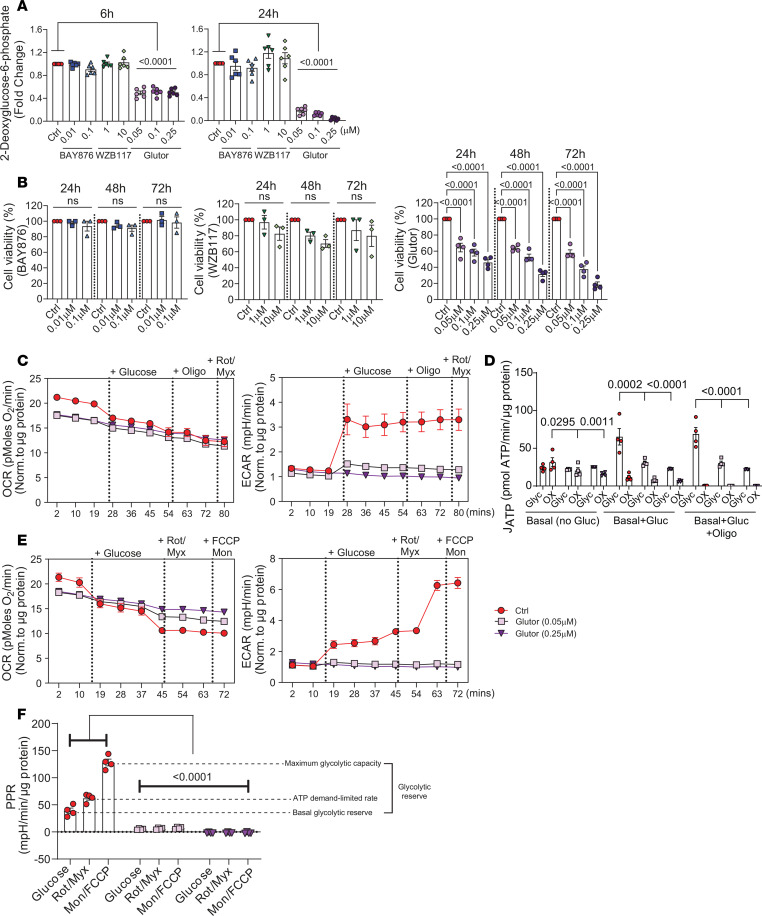
GLUT3 facilitates uptake of glucose, a metabolic substrate indispensable for NP cells. (**A**) 2-Deoxyglucose (2-DG) uptake in cells treated with GLUT1 inhibitors and GLUT1/3 dual inhibitor Glutor from 6 to 24 hours. (**B**) Cell viability following GLUT1 (BAY-876 and WZB-117) and GLUT1/3 inhibition (Glutor). (**C**) OCR and ECAR traces in 24-hour Glutor-treated and control NP cells to assess ATP production rate. (**D**) Inhibition of GLUT1 and GLUT3 by Glutor significantly decreased the ATP production by NP cells. (**E**) OCR and ECAR traces in 24-hour Glutor-treated and control NP cells to assess proton production rate. (**F**) Inhibition of GLUT1 and GLUT3 by Glutor significantly decreased the glycolytic reserve in NP cells. Quantitative measurements represent mean ± SEM (*n* = 4–6 biological replicates, 4 technical replicates/experiment/group). The significance of differences was determined using 1-way ANOVA with Holm-Šídák or Dunnett’s post hoc test as appropriate.

Although GLUT1 is the highest expressed transporter in the NP, other glucose transporters, including hypoxia-sensitive GLUT3 and GLUT9, are reported to be expressed in the NP and may also facilitate glucose uptake (3, 23). To test this hypothesis, we evaluated if GLUT1 is solely required for glucose import by measuring glucose uptake in NP cells treated with GLUT1 inhibitors, BAY-876/WZB-117, and the potent GLUT1/2/3 inhibitor, Glutor. We treated primary NP cells with the glucose mimic, 2-deoxyglucose (2-DG), and measured the subsequent intracellular accumulation of 2-deoxyglucose-6-phosphate, which cannot undergo glycolysis. The 2-DG uptake assay clearly showed that simultaneously blocking GLUT1 and GLUT3 decreased glucose uptake by up to 80% to 90% compared with control in a dose- and time-dependent manner (Figure 3A). Interestingly, GLUT1 inhibition alone for 6 and 24 hours with BAY-876 or WZB-117 did not result in decreased glucose uptake, implying a rapid compensation for loss of GLUT1 function. These findings also suggest that alternative glucose transporters, such as GLUT3 (NP cells do not express GLUT2, which has a high *K_m_* for glucose of ~17.1 mM), compensate for GLUT1 and may be responsible for much of the glucose uptake in NP cells. These results raised a possibility that simultaneous, long-term inhibition of GLUT1 and GLUT3 will compromise NP cell survival. We, therefore, measured NP cell viability following Glutor treatment for up to 72 hours. It was evident that cell viability decreased with increasing Glutor dose and the time of treatment (Figure 3B).

To ascertain if GLUT1/3 inhibition impacts NP cell metabolism, we performed Seahorse assays to measure ATP production rate and glycolytic flux of NP cells treated with 2 concentrations of Glutor for 24 hours (Figure 3, C–F). Glutor-treated NP cells did not show an increase in average ECAR and a corresponding decrease in average OCR following glucose addition and cotreatment with mitochondrial inhibitors as evidenced by untreated controls (Figure 3, C and E). Importantly, there was a significant decrease in glycolytic ATP production rate in Glutor-treated cells in the presence of glucose with or without oligomycin, which inhibits mitochondrial ATP generation (Figure 3D). Moreover, Glutor treatment resulted in a complete and striking collapse of glycolytic capacity and reserve compared with control cells (Figure 3F). These results clearly indicated that in NP cells, glucose uptake was sustained by both GLUT1 and GLUT3 and supported the notion that GLUT3 can compensate for GLUT1 loss.

**Figure 4:**
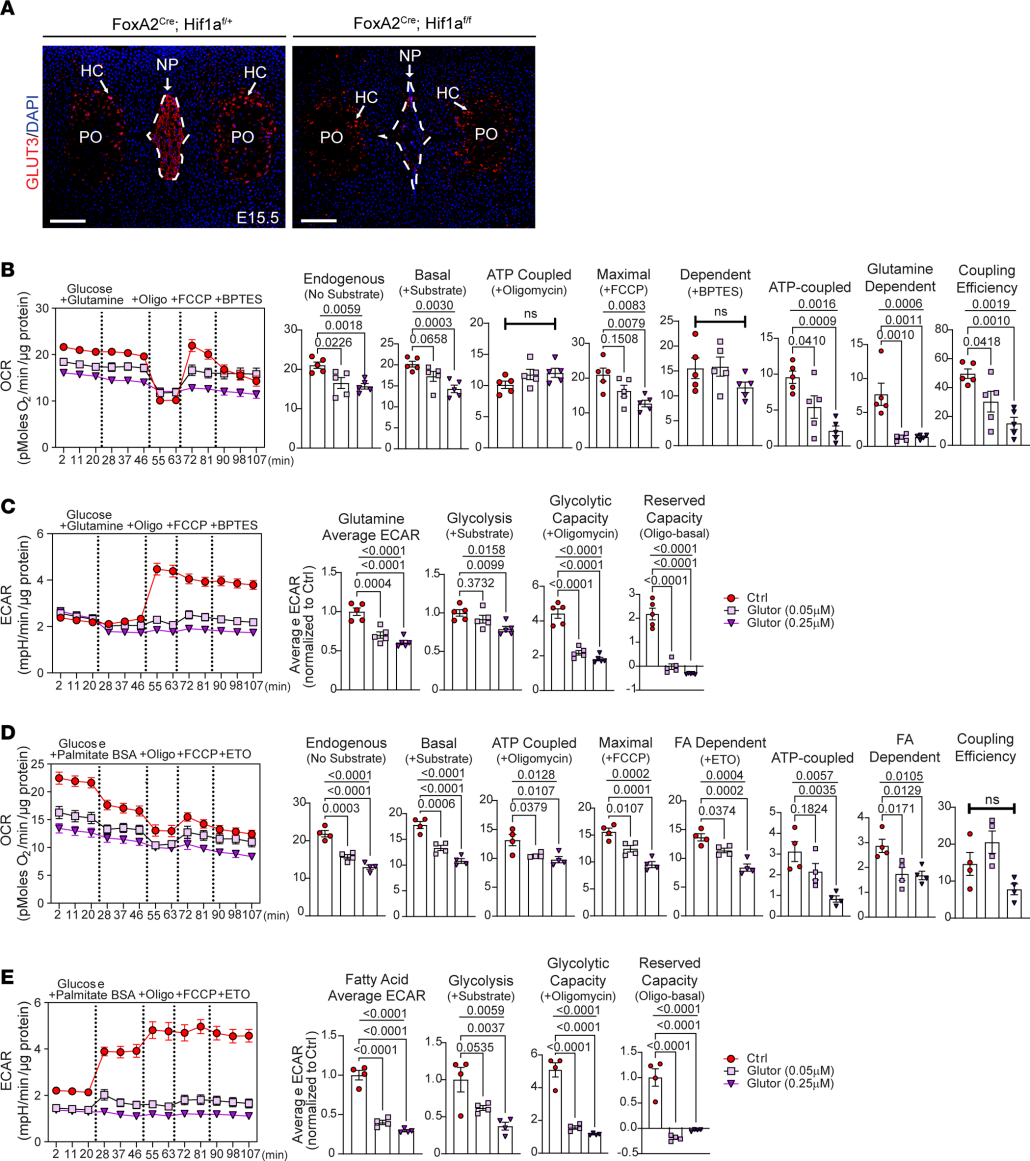
NP cells do not switch to glutamine utilization and fatty acid oxidation following loss of GLUT1 and GLUT3. (**A**) Representative image of GLUT3 staining in *Hif1a^fl/fl^* (WT) and *Hif1a*^cKO^ (*Foxa2^Cre^ Hif1a^fl/fl^*) mice (scale bar = 100 μm). PO, primary center of ossification; HC, hypertrophic chondrocytes. (**B**) OCR and (**C**) ECAR traces in Glutor-treated and control cells in presence of glutamine and BPTES. (**B**) Quantification of endogenous, basal, maximal, and glutamine-dependent OCR derived from traces and (**C**) glutamine-dependent average ECAR and glycolysis derived from traces. (**D**) OCR and (**E**) ECAR traces in Glutor-treated and control NP cells in presence of palmitate-BSA, and etomoxir. (**D**) Quantification of endogenous, basal, maximal, glutamine-dependent OCR from traces and (**E**) average ECAR and glycolysis derived from traces. Quantitative measurements represent mean ± SEM (*n* = 4–6 biological replicates, 4 technical replicates/experiment/group). The significance of differences was determined using 1-way ANOVA with Holm-Šídák or Dunnett’s post hoc test as appropriate.

Moreover, similar to GLUT1, there was a robust decrease in GLUT3 levels in the NP of E15.5 *Hif1a*^cKO^ mice, suggesting that pronounced cell death observed at birth in the NP compartment of these cKO mice could be in part due to constrained availability of glucose aggravating metabolic failure (9) (Figure 4A and Supplemental Figure 1A; supplemental material available online with this article; https://doi.org/10.1172/jci.insight.164883DS1). Likewise, we observed a strong age-dependent decrease in GLUT3 abundance in the NP compartment with a significant reduction noted at 24 months (Supplemental Figure 1, B–D). Furthermore, quantitative reverse transcription PCR (qRT-PCR) analysis showed a significant decrease in mRNA levels of *Glut3*, *Glut9*, and *Sglt1* with aging (Supplemental Figure 2A). Interestingly, however, a significant increase in *Glut1*/*Slc2a1* mRNA was noted at 24 months, suggesting a plausible compensation for the loss of GLUT1. Together these results suggest a correlation of glucose availability to age-dependent degeneration.

### NP cells do not switch to glutamine and fatty acid oxidation under glucose-limiting conditions following GLUT1/3 inhibition.

Since glucose is the major energy source for most cell types, it is plausible to consider that blocking its availability will shift cellular metabolism toward other substrates such as glutamine and/or fatty acids to maintain TCA cycle flux and ATP generation. Our previous studies have shown that although NP cells do not rely on oxidative phosphorylation for ATP production, the TCA cycle is intact and serves as a hub for the generation of metabolic intermediates used in broad biosynthesis reactions. In recent years, TCA cycle intermediates have also been shown to “moonlight” in the nucleus, where they engage in the epigenetic regulation of DNA and histone modifications (38).

To gain insight into whether the significant loss of glucose import through GLUT1 and GLUT3 upregulates glutamine metabolism or fatty acid oxidation, we performed substrate-dependent Seahorse experiments with glucose + glutamine (Figure 4, B and C) or BSA-palmitate (Figure 4, D and E). To assess effects on glutamine metabolism, NP cells were treated with Glutor for 24 hours prior to Seahorse assessment. We analyzed raw OCR and ECAR traces beginning with endogenous conditions (no substrate), followed by the sequential addition of glucose + glutamine (basal), oligomycin (ATP synthase inhibitor), FCCP (oxidative phosphorylation uncoupler), and BPTES (glutaminase inhibitor) (Figure 4, B and C). Glutor treatment significantly decreased endogenous OCR and basal OCR in the presence of glucose and glutamine, implying glutamine oxidation alone was not sufficient to rescue OCR in NP cells (Figure 4B). Subsequently, control and Glutor-treated NP cells showed an expected decrease in OCR with oligomycin treatment; however, FCCP was unable to shift 0.25 μM Glutor–treated cells to maximal OCR regardless of glutamine availability (Figure 4B). To determine the contribution of glutamine oxidation, we inhibited glutaminase with BPTES. Interestingly, BPTES treatment did not decrease OCR in the control group, suggesting that NP cells did not prefer glutamine oxidation in presence of glucose. Importantly, taken together these data indicate that blocking glucose import through GLUT1/3 did not result in increased glutamine oxidation in NP cells (Figure 4B). Similarly, in the presence of glucose and glutamine, Glutor-treated cells showed a decrease in average ECAR, whereas glycolysis-dependent ECAR was only affected at higher Glutor concentrations. Moreover, unlike control cells, Glutor-treated cells in presence of oligomycin (ETC inhibition) were unable to increase ECAR (Figure 4C). Of note, the presence of glutamine affected glucose-dependent ECAR in control cells; i.e., glucose addition did not increase ECAR, suggesting slowed glycolytic flux (Figure 4C). These data imply that glutamine is not sufficient to rescue cell metabolism in the absence of glucose and in fact may interfere with glucose use by NP cells.

Considering that GLUT1/3 inhibition did not cause NP cells to switch to glutamine oxidation, we investigated whether glucose restriction resulted in the use of fatty acids to maintain TCA cycle flux. For these experiments, OCR was measured during endogenous conditions (no substrate), followed by the sequential addition of substrate (glucose + palmitate-BSA), oligomycin, FCCP, and finally etomoxir (CPT1 inhibitor; inhibits mitochondrial import of fatty acid) (Figure 4D). Predictably, the endogenous OCR was decreased in Glutor-treated cells, and the addition of glucose + palmitate-BSA did not rescue OCR (Figure 4D). Moreover, inhibiting fatty acid import with etomoxir resulted in ~25% decrease in OCR in control cells and ~15%–25% decrease in Glutor-treated cells (Figure 4D). It was interesting to note that unlike glutamine, inclusion of BSA-palmitate with glucose did not interfere with ECAR induction in control cells (Figure 4E). However, palmitate did not increase ECAR in Glutor-treated cells, which remained significantly lower than in control cells (Figure 4E). Taken together, these metabolic experiments suggested that loss of GLUT1/3 function did not result in NP cells switching to glutamine or fatty acid oxidation and that glucose import through GLUT1/3 is indispensable for their metabolism.

### NP-specific deletion of GLUT1 in skeletally mature mice does not affect disc health.

**Figure 5:**
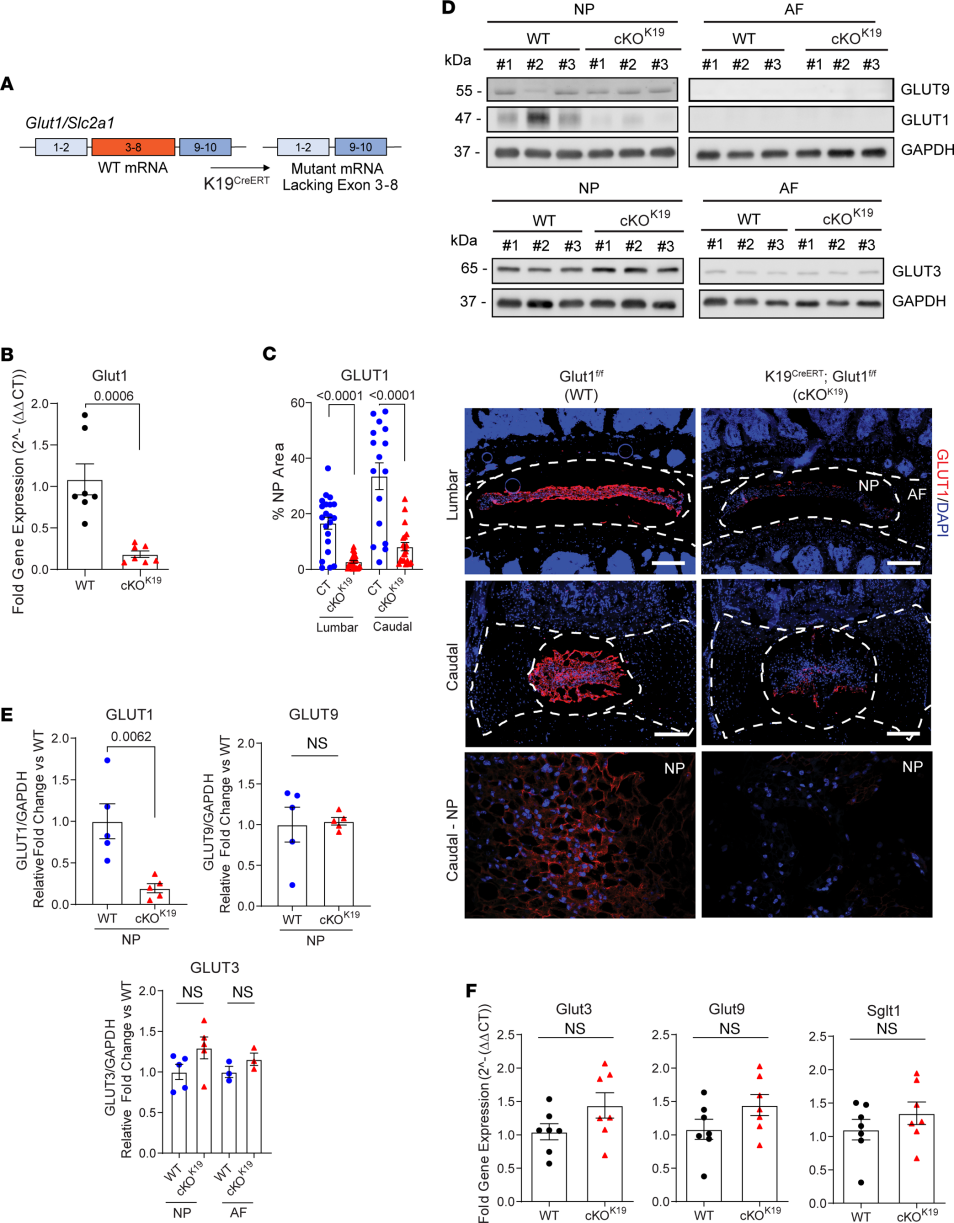
Conditional deletion of Glut1 in NP compartment of adult mice. (**A**) Schematic showing K19^CreERT^-mediated deletion of *Glut1/Slc2a1* exons 3–8 to generate NP-specific *Glut1* mutant. (**B**) Quantification of *Glut1* in cKO^K19^ mice (*n* = 7 mice/genotype; 20 discs/animal). (**C**) Representative IHC images and quantification of GLUT1 in 9-month-old WT and *Glut1*cKO^K19^ (scale bar = 200 μm and 50 μm) (*n* = 8 WT, 7 cKO mice; 6 lumbar and 3 caudal discs/animal). White dotted lines demarcate disc compartments. (**D** and **E**) Western blot and quantification of GLUT1 and GLUT9 levels in NP and AF tissues of WT and *Glut1*cKO^K19^ mice (*n* = 5 mice/genotype; 20 discs/animal); blots were first probed for GLUT1, stripped, and reprobed for GLUT9. Western blot and quantification of GLUT3 in NP and AF (*n* = 5 mice/genotype; 20 discs/animal). (**F**) qRT-PCR of *Glut3*, *Glut9*, and *Sglt1* in WT and *Glut1*cKO^K19^ mice (*n* = 7 mice/genotype; 20 discs/animal). Quantitative measurements represent mean ± SEM; the significance of differences was determined using Mann-Whitney *U* test.

To elucidate the role and test the apparent redundancy of GLUT1 seen in our in vitro experiments, we generated a conditional *Glut1* knockout in the mouse NP, driven by a tamoxifen-inducible Keratin-19 CreERT (*K19^CreERT^*) allele (Figure 5A). The *K19^CreERT^ Glut1^fl/fl^* (cKO^K19^) and littermate control *Glut1^fl/fl^* (WT) mice were injected with tamoxifen at 3 months (3M) and tissues collected at 9 months (9M). Robust *Glut1*/*Slc2a1* mRNA knockdown, approximately 80%–90%, was confirmed by qRT-PCR (Figure 5B). Deletion of GLUT1 protein was confirmed by fluorescence immunohistochemistry in both lumbar and caudal discs (Figure 5C) as well as Western blot (Figure 5, D and E, and Supplemental Figure 3A). These results validate the cKO^K19^ mouse model and validate NP-specific deletion of GLUT1 protein expression in skeletally mature mice.

We also verified the expression of alternative glucose transporters and determined if their levels are affected in cKO^K19^ mice. There were comparable levels of *Glut3* and *Glut9*, as well as sodium/glucose cotransporter *Sglt1*, mRNA between the WT and cKO^K19^ mice (Figure 5F). Furthermore, in line with previous findings in the human disc, Western blot analysis confirmed that GLUT3 and GLUT9 protein levels were robust in the NP of WT and cKO^K19^ mice, with significantly lower abundance in the AF (Figure 5, D and E, and Supplemental Figure 3, B and C) (23). Based on these findings, we hypothesized that baseline levels of alternative glucose transporters in the NP, including GLUT3, GLUT9, and SGLT1, may be sufficient to sustain glucose transport and preserve disc health in GLUT1-deficient mice.

**Figure 6:**
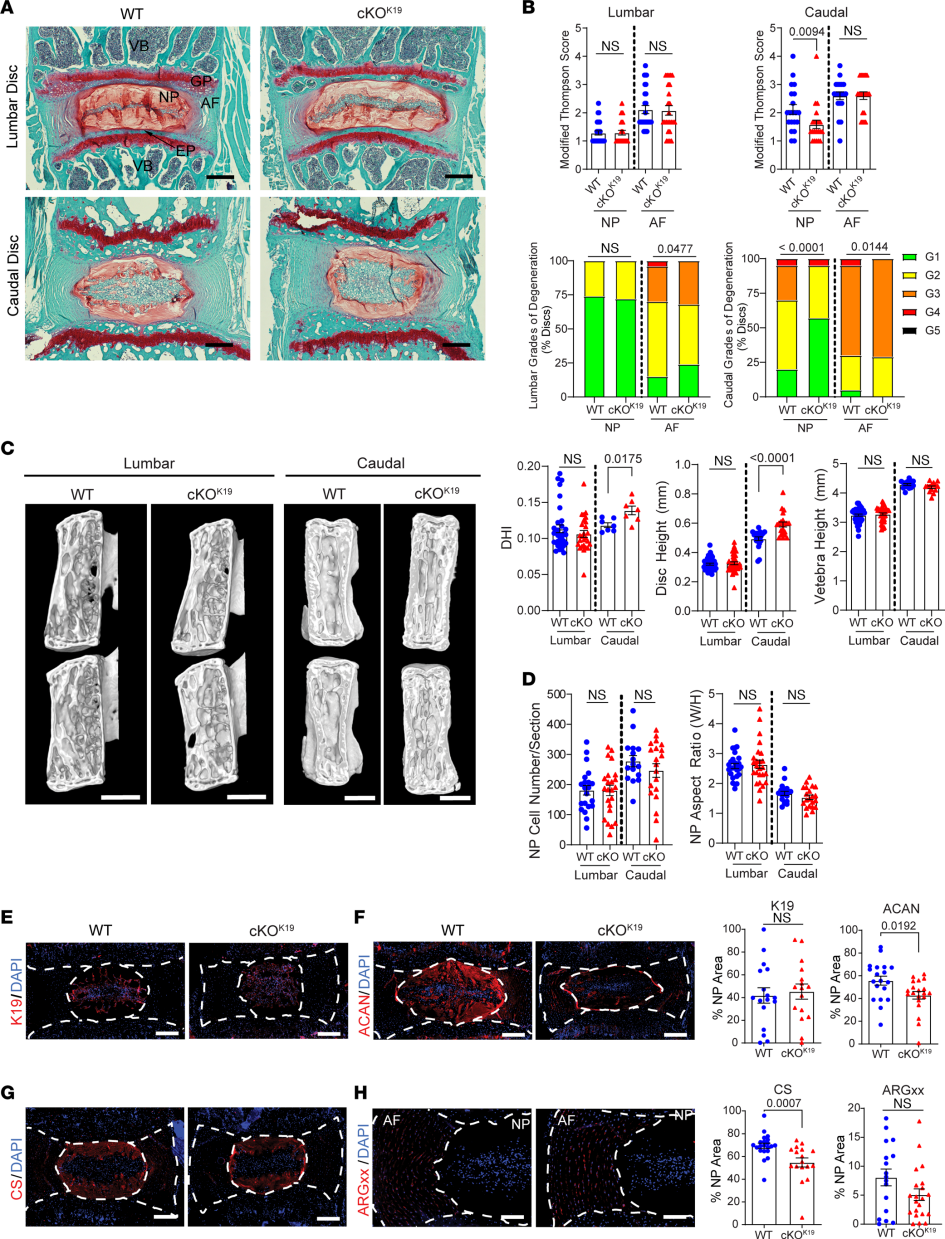
*Glut1*cKO^K19^ mice do not show adverse effects on intervertebral disc health. (**A**) Representative Safranin O/Fast Green images of 9-month-old WT and *Glut1*cKO^K19^ lumbar and caudal discs (scale bar = 200 μm). VB, vertebral body; GP, growth plate; EP, cartilaginous endplate. (**B**) Modified Thompson Scores of NP and AF compartments in WT and *Glut1*cKO^K19^ lumbar and caudal discs. (*n* = 7 mice/genotype; 3–4 lumbar and 2–3 caudal discs/animal, 25–27 lumbar and 20–21 caudal discs/genotype). (**C**) Representative μCT images of lumbar and caudal motion segments (scale bar = 1 mm). Quantification of lumbar and caudal DHI, DH, and vertebral body height (VBH) (*n* = 7 animals/genotype; vertebrae L3–6 and Ca5–6 and discs L3/4–L6/S1 and Ca5/6–7/8 per animal). (**D**) Quantification of NP cell number and aspect ratio from lumbar and caudal discs. (*n* = 7 mice/genotype; 1–4 lumbar discs/animal, 2–3 caudal discs/animal; 25–27 lumbar and 20–21 caudal discs/genotype.) (**E**–**H**) Representative IHC and quantification of K19, aggrecan (ACAN), chondroitin sulfate (CS), and ARGxx (neoepitope marker of cleaved ACAN) (scale bar = 200 μm, ARGxx scale bar = 100 μm). (*n* = 7 mice/genotype; 1–3 caudal discs/animal; 15–20 caudal discs/genotype.) White dotted lines demarcate disc compartments. The significance for grading distribution was determined using a χ^2^ test. The significance of differences was determined using an unpaired *t* test (**C**–**E**) or Mann-Whitney *U* test (**B**–**D** and **F**–**H**), as appropriate. Quantitative measurements represent mean ± SEM.

Histological changes were assessed in lumbar and caudal discs of WT and cKO^K19^ at 9M of age (Figure 6A). Despite the robust expression of GLUT1 in the NP of healthy discs, cKO^K19^ animals presented with no significant changes in the average grade of degeneration as measured by Modified Thompson Score (Figure 6B) or in the distribution in grades of degeneration (Figure 6B) in the NP of lumbar discs. There was a change in the distribution in grades of degeneration in the lumbar AF; however, it did not indicate increased degeneration. Curiously, caudal cKO^K19^ discs showed a slight decrease in the average grade of NP degeneration (Figure 6B), and distributions skewed toward lower grades of degeneration for NP and AF as well (Figure 6B). Taken together, NP-specific deletion of GLUT1 in adult mice had few discernible effects on disc degeneration in lumbar or caudal discs.

To evaluate the impact of the loss of GLUT1 on disc height (DH) and disc height index (DHI), μCT imaging was performed on 9M WT and KO^K19^ mice (Figure 6C). In line with the histological analysis, we observed no changes in either DH or DHI in lumbar discs; however, there was a slight increase in both metrics in caudal discs (Figure 6C). The increased DHI in caudal discs could not be accounted for by changes in the NP cell area (Figure 6D) or in the aspect ratio of the NP tissue compartment (Figure 6D).

To determine whether the cKO^K19^ mice show alterations in cell phenotypic markers and extracellular matrix (ECM) composition that are not reflected in histological grades of disc degeneration, we assessed the abundance of key ECM proteins in WT and cKO^K19^ discs. K19, an NP cell phenotypic marker, showed no difference in abundance, suggesting the NP cell phenotype was maintained in cKO^K19^ mice (Figure 6E). Interestingly, in the NP compartment of cKO^K19^ mice, there was a significant decrease in the level of aggrecan (ACAN) — the major NP proteoglycan — as well as chondroitin sulfate (CS) (Figure 6, F and G). To understand if the decrease in ACAN and its predominant glycosaminoglycan (GAG) chain was due to ACAN turnover, we quantified changes in a neoepitope marker of cleaved ACAN, ARGxx. Since there was no difference in ARGxx levels in discs of WT and cKO^K19^ mice, the results suggested a possible decrease in the synthesis of these molecules (Figure 6H). Accordingly, while adult cKO^K19^ mice showed negligible differences in overall disc morphology, alteration in GAG synthesis suggests a small deficit in glucose availability and increased glucose flux into lactate/pyruvate to maintain ATP levels likely affecting hexosamine biosynthetic pathway.

We also made note of the significant difference in the distribution of grades of degeneration in the AF compartment of both lumbar and caudal discs in cKO^K19^ mice (Figure 6B). To assess AF ECM, we analyzed collagen fiber architecture using Picrosirius red staining coupled with polarized light imaging (Supplemental Figure 4, A and B). Under polarized light, green fluorescing fibers are thin, yellow fluorescing fibers are intermediate, and red fluorescing fibers are thick. We found that collagen fiber thickness was unaltered in lumbar AF (Supplemental Figure 4A); however, in cKO^K19^ caudal discs, there was a significant increase in the percentage of thin (green) fibers and a decrease in thick (red) fibers (Supplemental Figure 4B). This suggests that NP-specific deletion of GLUT1 may alter collagen fibers in the AF, leading to a thinner and more immature fiber composition.

### Loss of GLUT1 does not alter the expression of genes involved in compensatory metabolic pathways.

To understand if disc health in cKO^K19^ mice is maintained by regulation of compensatory mechanisms, we analyzed the NP transcriptome using microarray. There was a similar variance between the gene expression values in NP cells from WT and cKO^K19^ mice, and the 2 phenotypes did not cluster independently along 3 principal components (Supplemental Figure 5A). When analyzed using a *P* value cutoff of *P* ≤ 0.05 and a fold-change of ±2, several differentially expressed genes emerged from the data set (Supplemental Figure 5, B and C), the most significant being *Slc2a1*/*Glut1*. However, PANTHER gene ontological analysis discovered no significantly enriched biological processes or molecular functions in the up- and downregulated gene sets (data not shown). Furthermore, when the data were analyzed using more stringent parameters — FDR ≤ 0.05 and fold-change of ± 2 — the only differentially expressed gene was *Glut1*/*Slc2a1* (Supplemental Figure 5D).

The microarray analysis clearly shows that loss of GLUT1 causes strikingly few transcriptional changes in NP cells. This finding suggests that compensatory metabolic pathways do not require alterations in gene expression, despite the strict dependence of NP cells on glucose uptake. Changes in flux through alternative glucose transporters may therefore be sufficient to maintain the glycolytic capacity in the NP of cKO^K19^ mice.

### GLUT1 expression in notochord/NP is not required for normal disc development.

**Figure 7:**
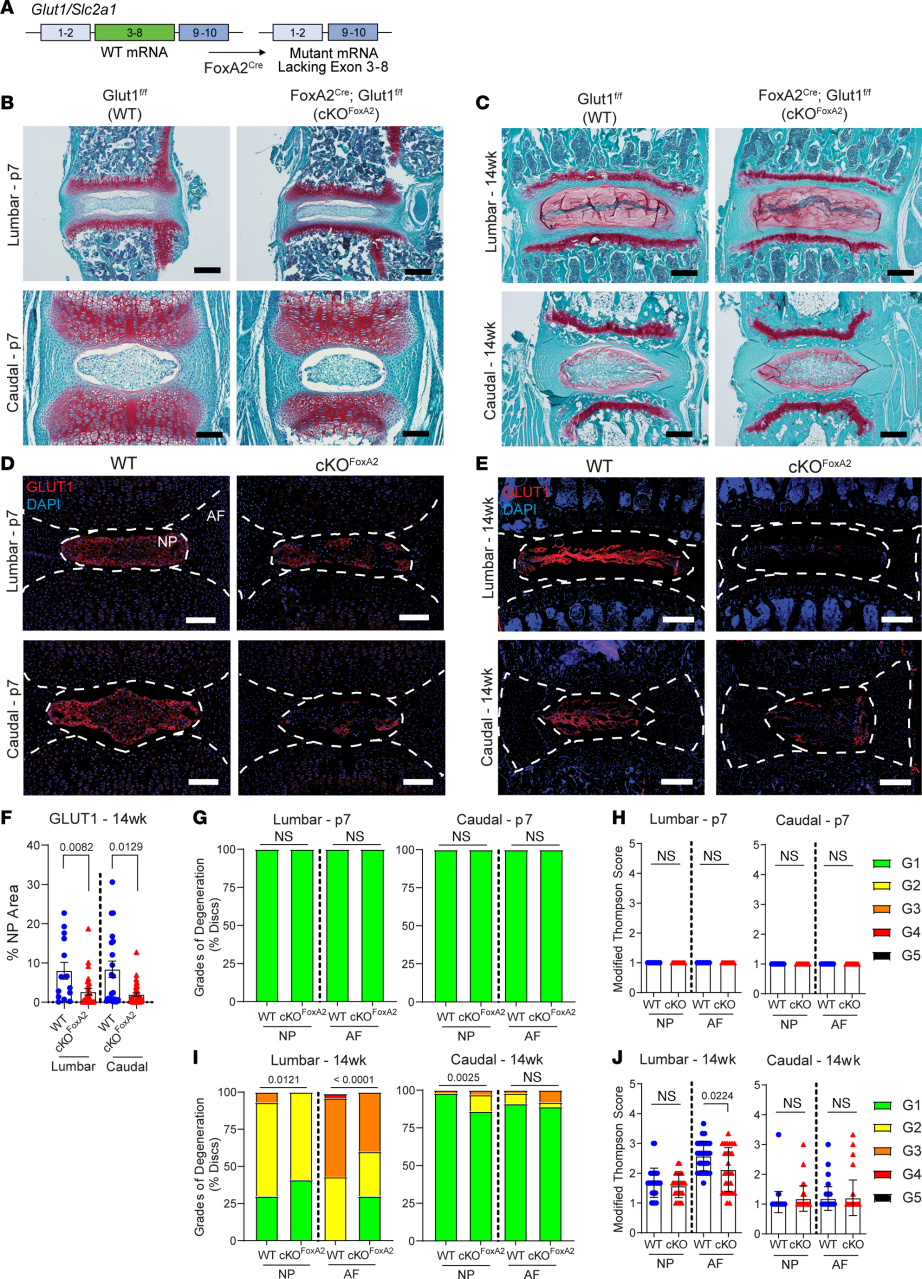
*Glut1*cKO^Foxa2^ mice do not show compromised disc health. (**A**) Schematic of *Foxa2-Cre*–mediated deletion of exons 3–8 in *Glut1^fl/fl^* mice to generate an NP-specific *Glut1*-cKO mice. (**B**–**E**) Representative images of (**B** and **C**) Safranin O/Fast Green staining and (**D** and **E**) GLUT1 IHC on *Glut1^fl/fl^* (WT) and *Foxa2^Cre^ Glut1^fl/fl^* (cKO^Foxa2^) discs at P7 and 14wk (P7 scale bar = 50 μm, 14wk scale bar = 200 μm). White dotted lines demarcate disc compartments. (**F**) Quantification of GLUT1 IHC at 14wk (*n* = 5 WT and 9 cKO^Foxa2^; 3 lumbar and 4 caudal discs/animal). (**G**) Modified Thompson grade distribution and (**H**) average grade scores of WT and cKO^Foxa2^ discs at P7 (*n* = 5 mice/genotype; 6 discs: 3 lumbar and 3 caudal/animal). (**I**) Modified Thompson grade distribution and (**J**) average grade scores of WT and cKO^Foxa2^ discs at 14wk (*n* = 10 WT and 10 cKO^Foxa2^; 3 lumbar and 4 caudal/animal). The significance of grade distribution was determined using a χ^2^ test. The significance of differences in average grade scores and percentage staining area was determined using Mann-Whitney *U* test. Quantitative measurements represent mean ± SEM.

Considering that GLUT1 deletion in skeletally mature mice had no consequential effect on intervertebral disc health, we tested whether there was a temporal dependency on GLUT1 function, specifically, if it was required for normal embryonic and perinatal development of the NP. We crossed *Glut1^fl/fl^* mice with notochord and floor plate–specific constitutive *Foxa2^Cre^* mice to generate notochord/NP-specific GLUT1-KO mice — *Foxa2^Cre^ Glut1^fl/fl^* (i.e., cKO^Foxa2^) — and littermate control (WT) mice (Figure 7A) and aged them to postnatal day 7 (P7) and 14 weeks (14wk), when their morphology was assessed (Figure 7, B and C).

Immunohistochemistry verified a substantial decrease in GLUT1 expression in the NP compartment of cKO^Foxa2^ mice at P7 (Figure 7D) and 14wk (Figure 7E). Despite the substantial GLUT1 knockdown (Figure 7F), lumbar and caudal discs from cKO^Foxa2^ mice did not present with any of the criteria for degeneration; in fact all NP and AF compartments from P7 mice had a Modified Thompson Score of 1 (Figure 7, G and H). At 14wk, the distribution in the grades of degeneration in cKO^Foxa2^ was different from the WT in lumbar NP and AF, as well as caudal NP; however, the distributions favored lower grades of degeneration in the cKO^Foxa2^ mice (Figure 7I). Consequently, cKO^Foxa2^ mice lumbar AF had a slightly lower Modified Thompson Score, implying better morphological attributes, while lumbar NP and caudal NP and AF showed no significant difference from WT (Figure 7J). These results suggest that glucose uptake through GLUT1 is not a critical player for the development and early maintenance of the NP.

## Discussion

**Figure 8:**
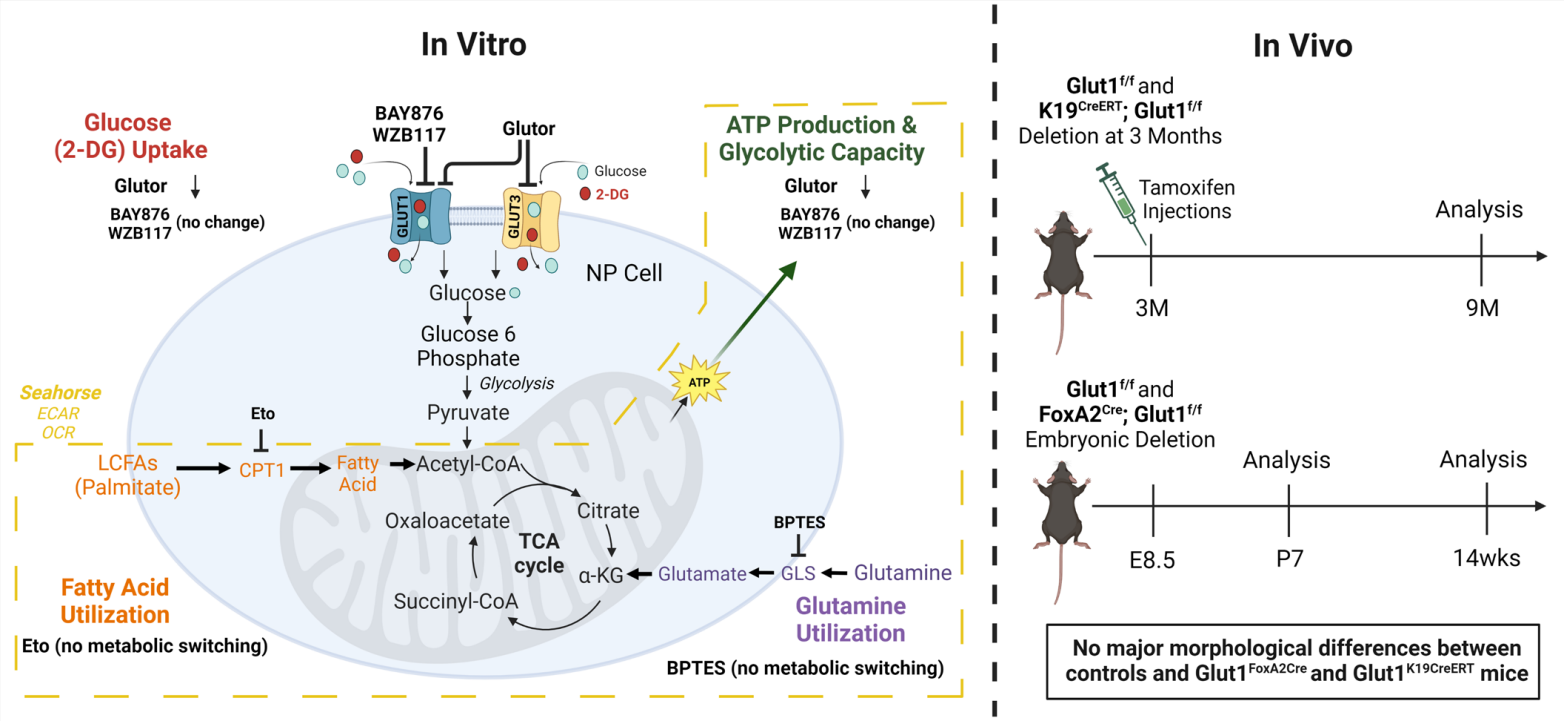
A schematic summarizing the in vitro experimental approaches and their outcomes and in vivo mouse models to test the role of GLUT1 in maintaining NP tissue metabolism. Created with BioRender.com.

The fact that loss of GLUT1 expression in the disc does not result in disc degeneration, transcriptomic changes, or metabolic disruption was baffling, especially considering the prominence of GLUT1 as a highly enriched NP phenotypic marker and the requirement for GLUT1 in the functional maintenance of other skeletal tissues, including bone and cartilage (27, 28, 31). Indeed, our study underscores that although glucose is the indispensable metabolite, it appears that GLUT1 is not singularly required to maintain glycolytic capacity, and therefore, is not necessary for NP cell survival (Figure 8). Conversely, we hypothesize that maintaining ATP levels through glycolysis and TCA metabolites is so vital for disc health and NP cell viability that there is intrinsic redundancy to ensure an uninterrupted supply of glucose through glucose importers other than GLUT1.

In the hypoxic niche of the intervertebral disc, NP cells primarily rely on glycolysis for bioenergetics and highly express GLUT1, which is considered one of their key phenotypic markers. We therefore investigated the role GLUT1 plays in NP cell metabolism by using 2 highly potent GLUT1 inhibitors (35, 36). Surprisingly, since blocking GLUT1 in NP cells did not affect glucose uptake and the bioenergetic status of cells, the results suggested an alternative mechanism for glucose import. This finding was in contrast to bone cells that primarily depend on glucose for both differentiation and maturation during development and highly express GLUT1, which is responsible for 75% of glucose uptake (27). One obvious explanation for this difference is that we and others have previously shown that NP cells express GLUT3 and GLUT9 (8, 23). Using a dual GLUT1/3 inhibitor, Glutor, we determined that 80%–90% glucose was imported through these 2 carriers (39). Importantly, unlike GLUT1 inhibition, Glutor treatment significantly inhibited glycolysis and diminished ATP production rate, suggesting that GLUT3 is the critical high-capacity glucose importer in NP cells. This also implies that NP cells in their avascular and diffusionally limited environment are intrinsically geared to import and utilize glucose as a metabolic substrate in a broad physiological concentration range given the diverse *K_m_* values for GLUT1 (~6.9 mM), GLUT3 (1.8 mM), and GLUT9 (0.61 mM) (40, 41).

It is known that under nutritional constraints or increased metabolic demand, cellular function and survival are mediated by metabolic reprogramming (30, 42, 43). When glucose uptake is blocked, primarily glycolytic proinflammatory macrophages and cancer cells switch their metabolism to alternative substrates such as glutamine and fatty acids (30, 42). Similarly, a very recent study showed that deletion of GLUT1 in articular and growth plate chondrocytes resulted in increased cellular glutamine oxidation for survival (31). Similarly, in the absence of glucose, osteoblasts shift to glutamine but not palmitate oxidation, whereas myoblasts prefer palmitate oxidation over glutamine oxidation (27). It is important to note that NP cells also exhibit metabolic plasticity. For example, when lactate export through monocarboxylate transporter MCT4 is inhibited in NP cells, the cells undergo an incomplete metabolic switch from glycolytic to oxidative metabolism fueled by pyruvate oxidation (16). However, blocking lactate export still results in a 2-fold increase in the glycolytic intermediate, glucose-6-phosphate, implying glucose was still the major metabolite fueling pyruvate metabolism (16). While NP cells do have the capacity to metabolize alternative energy sources, such as fatty acids through mitochondrial β-oxidation (8), many studies including our own show that glucose starvation leads to cell death (12, 14, 22). Therefore, alternative energy sources such as glutamine, glycogen, and fatty acids cannot compensate for the loss of glucose import into NP cells. Furthermore, in support of this notion, we noted that when glucose import was impeded by GLUT1/3 inhibition, NP cells did not shift to glutamine or palmitate oxidation. Rather, in contrast to other cell types, the overall OCR significantly decreased, underscoring the fact that glucose is the primary metabolic fuel for ATP generation through glycolysis and is critical for NP cell survival (8, 39).

We have previously shown that in SM/J mice, a mouse model of early-onset spontaneous degeneration, GLUT1 levels in the NP decline, and as our current studies show, levels are lower during aging, an important risk factor associated with disc degeneration (33, 44, 45). These findings seemingly contradict our in vitro findings that showed minimal or no change in NP cell metabolism and their survival following GLUT1 inhibition. To clarify this apparent conflict and to delineate whether loss of GLUT1 expression in vivo causes disc degeneration, we used 2 mouse models, an inducible *Glut1*cKO^K19^ and constitutive *Glut1*cKO^Foxa2^, to determine a causal link between GLUT1 and intervertebral disc health. While there were some differences in grades of degeneration, between these mice, the cKO^K19^ mice rather showed slightly lower grades of degeneration. However, that is not to say that GLUT1 loss of function is protective in the disc, but rather the discs are generally healthy in both WT and cKO animals. In a parallel study, we used a constitutive cKO^Foxa2^ model to determine if GLUT1 is involved in disc development and postnatal maturation. This hypothesis stems from the idea that GLUT1 may play a key role in the HIF1α-dependent glycolytic metabolism in the developing embryonic NP (9). The lack of apparent developmental defects in the discs from these mice further suggested that NP cells are refractory to loss of GLUT1 and that avenues of compensation are available to NP cells to allow them to survive the deletion of an integral glycolytic component. However, it may be of interest to ascertain whether functional compensation of GLUT1 or GLUT3 occurs when additional systemic stressors such as acute injury or aging are involved. Importantly, however, these observations raised an interesting prospect that a robust decrease in GLUT3 levels along with GLUT1 may contribute to metabolic failure and massive cell death of NP cells seen in *Hif1a*^cKO^ mice by birth (9). Likewise decline in both GLUT3 and 1 may exacerbate metabolic restriction on cells in the aging disc, in part driving loss of cells and an overall decreased biosynthesis of matrix molecules, leading to age-dependent degeneration (1).

Another key observation was that despite GLUT1 loss of function, compensatory pathways do not involve transcriptional upregulation of other glucose transporters or metabolic enzymes. In fact, microarray analysis of NP from cKO^K19^ mice, when analyzed with an adjusted FDR *P* value of ≤ 0.05, failed to reveal a single differentially regulated transcript besides *Glut1/Slc2a1*. These data are corroborated by the lack of changes in mRNA expression or protein levels of other *SLC2* family members and glucose importers expressed in the disc, namely GLUT3 and GLUT9 (23). Importantly, however, glucose import may be maintained in NP cells by increased flux through these SLC2 transporters without concomitant elevation in expression levels. Furthermore, glucose may also enter the NP cell through sodium-glucose cotransport (SGLT), of which 6 isoforms have been identified (46). SGLT is driven by the active extrusion of intracellular sodium, facilitating a concomitant import of extracellular glucose against plasma-membrane concentration gradients. Although SGLTs are attributed to glucose import across apical membranes, it is very possible they play a role in the NP compartment, which is characterized by its high sodium concentrations in a hyperosmolar niche. While the contribution of these transporters has not yet been studied in the context of the intervertebral disc, it is likely to be secondary to SLC2 family members.

Taken together, our study provides the first evidence to our knowledge of functional redundancy in GLUT transporters in a physiologically hypoxic NP compartment of the intervertebral disc and highlights its uniquely different niche from other skeletal tissue like articular and growth plate cartilage (Figure 8). Importantly, our findings underscore the importance of glucose as the indispensable metabolic fuel for NP cells and provide a vital baseline for any cell-based therapies aimed at restoring the function of the degenerating disc.

## Methods

### Mice.

All procedures regarding collection of animal tissues were performed as per approved protocols by the Institutional Animal Care and Use Committee (IACUC) of Thomas Jefferson University, in accordance with the IACUC’s relevant guidelines and regulations. For postnatal deletion in NP compartment, *Glut1^fl/fl^* mice (47) were crossed with *K19^CreERT^* mice [*Krt19^tm1(cre/ERT)Ggu^*/J, The Jackson Laboratory stock 026925] to produce *K19^CreERT^ Glut1^fl/fl^* (cKO^K19^) mice, for which *Glut1^fl/fl^* littermate mice served as control (WT), and were injected with 3 consecutive tamoxifen injections at 3 months at a 100 μg/g BW; these mice were collected 6 months postrecombination (9 months old) (*n* = 8 *Glut1^fl/fl^* mice: 5 males, 3 females; and 7 cKO^K19^ mice: 3 males, 4 females) (48). For deletion of *Glut1* at embryonic time points, *Glut1^fl/fl^* mice were crossed with *Foxa2^Cre^* mice to produce *Foxa2^Cre^ Glut1^fl/fl^* (cKO^Foxa2^) mice, for which littermate *Foxa2^Cre^ Glut1^fl/+^* and *Glut1^fl/fl^* mice served as control (WT); these mice were assessed at P7 (*n* = 5 mice/genotype), 14wk (*n* = 10 mice/genotype; 5 female WT, 5 male WT, 4 female cKO^Foxa2^, 6 male cKO^Foxa2^). *Foxa2^Cre^* allele drives robust expression specifically in the notochord and floor plate using a combination of 5′ notochord and 3′ floor plate enhancers under the control of *Hspa1* promoter (49).

### Immunofluorescence microscopy and digital image analysis.

Midcoronal 7 μm disc sections were deparaffinized and incubated in microwaved citrate buffer for 20 minutes or proteinase K (New England BioLabs) for 10 minutes at room temperature or chondroitinase ABC (MilliporeSigma) for 30 minutes at 37°C for antigen retrieval. Appropriate WT and cKO histological sections were blocked in 5% normal serum (Normal Goat Serum, Thermo Fisher Scientific catalog 10000C, and Normal Donkey Serum, Jackson ImmunoResearch catalog 017-000-121) in PBS-T (0.4% Triton X-100 in PBS) and incubated with antibodies against KRT19 (1:3, DSHB, TROMA-III/supernatant), CA3 (1:150, Santa Cruz Biotechnology, sc-50715), ACAN (1:50, MilliporeSigma, AB1031), GLUT1 (1:200, Abcam, ab40084), ARGxx (1:200, Abcam, ab3773), and GLUT3 (1:200, Proteintech, 20403-1-AP) in blocking buffer at 4°C overnight. For GLUT1 (1:200, Abcam, ab40084) and ARGxx (1:200, Abcam, ab3773) staining, Mouse on Mouse Kit (Vector Laboratories, BMK2202) was used for blocking and primary antibody incubation. Tissue sections were thoroughly washed and incubated with Alexa Fluor 594–conjugated (excitation 591 nm, emission 614 nm) secondary antibodies (Jackson ImmunoResearch, anti-rabbit 711-585-152, anti-mouse 715-585-150, anti-goat 305-585-003), at a dilution of 1:700 for 1 hour at room temperature in the dark. The sections were washed again with PBS-T (0.4% Triton X-100 in PBS) and mounted with ProLong Gold Antifade Mountant with DAPI (Thermo Fisher Scientific, P36934). All mounted slides were visualized with Axio Imager 2 (Carl Zeiss) using 5×/0.15 NAchroplan (Carl Zeiss), 10×/0.3 EC Plan-Neofluar (Carl Zeiss), or 20×/0.5 EC Plan-Neofluar (Carl Zeiss) objectives; X-Cite 120Q Excitation Light Source (Excelitas); AxioCam MRm camera (Carl Zeiss); or LSM800 (Carl Zeiss) 20×/0.8 or 40×/1.3 Oil Plan-Apochromat (Carl Zeiss), AxioCam 506 mono (Carl Zeiss), and Zen2 software (Carl Zeiss). DAPI-positive cells were analyzed to assess cell number in disc compartments. All quantifications were done in 8-bit grayscale using the Fiji package of ImageJ (NIH). Images were thresholded to create binary images, and NP and AF compartments were manually segmented using the Freehand Tool. These defined regions of interest were analyzed either using the Analyze Particles (cell number quantification) function or using the Area Fraction measurement.

### Protein extraction and Western blotting.

Mouse NP tissue was carefully separated from AF tissue under a dissecting microscope (Carl Zeiss Stemi 305 stereo zoom microscope 0.8×–4.0×) as described previously (50–54). Following NP tissue extraction from WT and cKO^K19^ mice, cells were washed on ice with ice-cold 1× PBS with a protease inhibitor cocktail (Thermo Fisher Scientific). Cells were lysed with lysis buffer containing 1× protease inhibitor cocktail (Thermo Fisher Scientific), NaF (4 mM), Na_3_VO_4_ (20 mM), NaCl (150 mM), β-glycerophosphate (50 mM), and DTT (0.2 mM). Total protein (35 μg per sample) was resolved on 10% SDS-PAGE and transferred to PVDF membranes (Thermo Fisher Scientific). Membranes were blocked with 5% nonfat dry milk in TBST (50 mM Tris pH 7.6, 150 mM NaCl, 0.1% Tween 20) and incubated overnight at 4°C in 5% nonfat dry milk in TBST with anti-GLUT3 (1:500, Proteintech, 20403-1-AP) or anti-GLUT9 (1:500, Abcam, ab223470) antibodies. Specificity of all antibodies has been validated by the manufacturers using siRNA or negative control IgG. Immunolabeling was detected using ECL reagent and imaged using LAS4000 system (GE Life Sciences). Densitometric analysis was performed using ImageJ. All quantitative data are represented as mean ± SEM, and *n* = 2–5 animal/genotype.

### Isolation of rat NP cells, hypoxic culture, and cell treatments.

Rat NP cells were isolated as reported previously by Risbud and colleagues (5). Rat NP cells were maintained in DMEM supplemented with 10% FBS and antibiotics. Cells were cultured in a Hypoxia Work Station (Invivo2 400) with a mixture of 1% O_2_, 5% CO_2_, and 94% N_2_. To investigate the effect of GLUT inhibition, NP cells were treated with 1) a cocktail of GLUT1 inhibitors (BAY-876, 0.01, 0.1 μM, Cayman, 19961; or WZB-117, 1, 10 μM, Cayman, 19900) for 1 to 72 hours or 2) a GLUT1, 2, and 3 inhibitor (Glutor, 0.05, 0.1, and 0.25 μM; Sigma, SML2765) for 6 to 72 hours. Viability measurements following treatment of NP cells in hypoxia with BAY-876, WZB-117, and Glutor for 24–72 hours were performed using a Calcein AM cell viability assay as per manufacturer’s instructions (Invitrogen, C3100MP). All in vitro experiments were performed in hypoxia (1% O_2_) at least 3–6 independent times with 4 replicates/experiment/group and data represented as mean ± SEM.

### Seahorse XF analyzer respiratory assay.

The oxygen ECAR and OCR were measured using a method reported by Mookerjee and colleagues (37). Briefly, rat NP cells were plated in a 24-well Seahorse V7-PS assay plate and cultured for 36 hours in normoxia conditions. At 24 hours prior to assay, cells were treated with GLUT1 inhibitors BAY-876 or WZB-117 and cultured under hypoxia. Prior to measurement cells were washed 3 times with 500 μL of Krebs Ringer Phosphate HEPES (KRPH) and incubated at 37°C for 1 hour under 100% air. OCR and ECAR were measured by the addition of 10 mM glucose, 2 μg oligomycin, and 1 μM rotenone plus 1 μM myxothiazol. The rates of oxygen consumption and extracellular acidification were normalized to the protein content of the appropriate well. For the substrate dependency assay, 24 hours prior to assay, cells were treated with GLUT1/3 inhibitor Glutor and cultured under hypoxia. Prior to measurement cells were washed 3 times with 500 μL of KRPH and incubated at 37°C for 1 hour under 100% air. OCR and ECAR were measured by the addition of 10 mM glucose plus 4 mM glutamine, 2 μg oligomycin, 1 μM FCCP, and 5 μM BPTES for glutamine oxidation. For fatty acid oxidation, KRPH was supplemented with 0.5 mM l-carnitine and incubated at 37°C for 1 hour under 100% air. OCR and ECAR were measured by the addition of 10 mM glucose plus palmitate-BSA (palmitate concentration 150 μM), 2 μg oligomycin, 1 μM FCCP, and 5 μM etomoxir. The rates of oxygen consumption and extracellular acidification were normalized to the protein content of the appropriate well.

### 2-DG uptake assay.

Rat NP cells (20,000 cells/well) were seeded in a 96-well plate in a complete medium and were grown for 48 hours in normoxia. After 48 hours cells were treated with glucose transporter inhibitors for 6 and 24 hours under hypoxia. The 2-DG uptake was measured following the protocol (Abcam, ab136956). Briefly, adherent cells were washed 3 times with freshly prepared KRPH buffer (20 mM HEPES, 5 mM KH_2_PO_4_, 1 mM MgSO_4_, 1 mM CaCl_2_, 136 mM NaCl, 4.7 mM KCl, pH 7.4, 2% BSA). 2-DG (1 μM) was added to the cells together with the respective inhibitors in KRPH buffer and incubated for 40 minutes. DMSO without 2-DG served as control. After 40 minutes the 2-DG uptake was stopped by removing the assay buffer and washing 3 times with ice-cold KRPH buffer. Cell lysis and endogenous NAD(P) degradation were performed by adding extraction buffer to the cells and incubating for 30 minutes at 85°C. Cell lysates were kept on ice for 5 minutes, and neutralization was performed by adding a neutralization buffer. Then 2-DG uptake was measured following the protocol. All quantitative data are represented as mean ± SEM, and *n* = 6 independent experiments, 4 replicates/experiment/group.

### qRT-PCR analysis.

NP tissue was microdissected from 9M WT and cKO^K19^ animals. Tissues from L1/2–L6/S1 and Ca1/2–Ca14/15 of the same mouse were pooled and served as a single sample stored into RNAlater Reagent (Invitrogen) for minimum 2 days at –80°C (*n* = 7 mice/genotype; 20 discs/animal). Samples were homogenized with a Pellet Pestle Motor (Sigma-Aldrich, Z359971), and RNA was extracted using RNeasy Mini Kit (QIAGEN). RNA was quantified on a NanoDrop ND-100 spectrophotometer (Thermo Fisher Scientific). Purified RNA was then converted to cDNA using EcoDry Premix (Clontech). Gene-specific primers (IDT) and the template cDNA were added to Power SYBR Green Master Mix (Applied Biosystems). Primer sets were designed by IDT (Supplemental Table 1). Quantification of expression was done by the QuantStudio3 Realtime PCR system (Applied Biosystems) using ΔΔCT method and *Hprt* to normalize gene expression.

### Mouse histological analysis.

Mouse spines were harvested and fixed in 4% paraformaldehyde (PFA) for 24–48 hours and decalcified in EDTA (12.5%–20%) at 4°C for 15–21 days prior to paraffin embedding. Midcoronal 7 μm disc sections (Ca5/6–Ca8/9, L1/2–L6/S1) were stained with Safranin O/Fast Green/Hematoxylin or Picrosirius red, then visualized using a light microscope (Axio Imager 2, Carl Zeiss) or a polarizing microscope (Eclipse LV100 POL, Nikon). Histopathological grading was performed on *n* = 5 mice/genotype with 6 discs per mouse (30 discs/genotype) at P7 and 14wk (WT and cKO^Foxa2^), and *n* = 8 mice/genotype with 6 discs per mouse (48 discs/genotype) at 9M (WT and cKO^K19^). Modified Thompson grading was used to score NP and AF compartments by 3 graders under a blinded protocol. The aspect ratio of NP was determined by width divided by height of the NP tissue measured from Safranin O/Fast Green staining images of midcoronal tissue sections from 9-month-old WT and cKO^K19^ animals, and *n* = 6 mice/genotype with 3 discs per mouse (18 discs/genotype) using ImageJ software.

### μCT analysis.

μCT scans (Bruker, Skyscan 1275) were performed on WT and cKO^K19^ spines fixed with 4% PFA. Lumbar spine segments incorporating L2–S1 (7 mice/genotype) were scanned with an energy of 50 kV at 200 μA, resulting in a 15 μm^3^ voxel size resolution. Intervertebral disc height and vertebral length were measured and averaged along the dorsal, midline, and ventral regions in the sagittal plane, and DHI was calculated as previously described (44, 50–52, 55).

### Transcriptomic analysis and enriched pathways.

RNA was quantified on a NanoDrop ND-100 spectrophotometer, followed by RNA quality assessment analysis on a 2200 TapeStation (Agilent Technologies). Fragmented biotin-labeled cDNA (from 100 ng of RNA) was synthesized using the GeneChip WT Plus kit according to ABI protocol (Thermo Fisher Scientific). GeneChips, Mouse Clariom S, were hybridized with 2.5 μg fragmented and biotin-labeled cDNA in 100 μL of hybridization cocktail. Arrays were washed and stained with GeneChip hybridization wash & stain kit using GeneChip Fluidic Station 450. Chips were scanned on an Affymetrix GeneChip Scanner 3000 7G, using Command Console Software. Quality control of the experiment was performed by Expression Console Software v 1.4.1. Chp files were generated by sst-rma normalization from the Affymetrix.cel file using Expression Console Software. The experimental group was compared with the control group by using Transcriptome Analysis Console 4.0 (Thermo Fisher Scientific) and DEGs with a fold-change ± 2, *P* or FDR < 0.05. PANTHER tool was used to compute enriched pathways in DEGs that were altered with a fold-change ± 2, *P* < 0.05. For evaluating highly expressed genes in the developing notochord/NP and NP of healthy adult mice, data deposited by Peck et al., Gene Expression Omnibus GSE100934 (32), and Novais et al., Gene Expression Omnibus GSE134955 (33), were used.

### Data availability.

All data generated or analyzed during this study are included in this published article (and its supplemental files) or deposited in the Gene Expression Omnibus database (accession number GSE208396).

### Statistics.

Quantitative data are presented as mean ± SEM, data distribution was checked with Shapiro-Wilk normality test, and unpaired 2-tailed *t* test or Mann-Whitney *U* test was used as appropriate. Comparisons between more than 2 groups were performed by the 1-way ANOVA or Kruskal-Wallis test with appropriate post hoc analyses (Dunn’s or Dunnett’s or Holm-Šídák multiple comparisons test) using Prism 9 (GraphPad Software); *P* < 0.05. For histopathological analysis showing percentage degenerated discs and Picrosirius red percentage of AF area, χ^2^ test was used. A *P* value less than 0.05 was considered significant.

### Study approval.

All procedures regarding collection of animal tissues were performed as per approved protocols by the IACUC of Thomas Jefferson University, in accordance with the IACUC’s relevant guidelines and regulations.

## Author contributions

ESS, SNJ, MVR, and IMS designed the project. SNJ, VM, ESS, and DHN performed all experiments. ESS, SNJ, VM, IMS, and MVR wrote and edited the manuscript.

## Supplementary Material

Supplemental data

## Version 1 03/14/2023

In-Press Preview

## Version 2 04/24/2023

Electronic publication
