# Supplementary material for: GLUT1 is redundant in hypoxic and glycolytic nucleus pulposus cells of the intervertebral disc
Source: JCI Insight. 2023 Apr 24;8(8):e164883. doi: 10.1172/jci.insight.164883 (PMC10243741; doi:10.1172/jci.insight.164883)
Supplement: Supplemental data [file jciinsight-8-164883-s133.pdf]

Supporting Information for

GLUT1 is redundant in hypoxic and glycolytic nucleus pulposus cells of the intervertebral disc

Shira N. Johnston[1,2,#], Elizabeth S. Silagi[1,2,3,#], Vedavathi Madhu[1], Duc H. Nguyen[1,2], Irving M. Shapiro[1,2], Makarand V. Risbud[1,2,*]
[1]Department of Orthopaedic Surgery, Sidney Kimmel Medical College, Thomas Jefferson University, Philadelphia, PA, USA
[2]Graduate Program in Cell Biology and Regenerative Medicine, Jefferson College of Life Sciences, Thomas Jefferson University, Philadelphia, PA, USA
[3]Current affiliation - Department of Neuroscience, Dana Farber Cancer Institute, Harvard Medical School, Boston, MA, USA
[#] Have contributed equally to this work

Corresponding Author
Makarand V. Risbud, Ph.D.
James J. Maguire Jr. Professor of Spine Research, Orthopaedic Surgery
Division Director, Orthopaedic Research
Co-Director, Cell Biology & Regenerative Medicine Graduate Program
1015 Walnut Street
Suite 501, Curtis Bldg.
Philadelphia, PA 19107
Tel: 215-955-1063
Email: makarand.risbud@jefferson.edu

This PDF file includes:

Figures S1 to S5
Tables S1

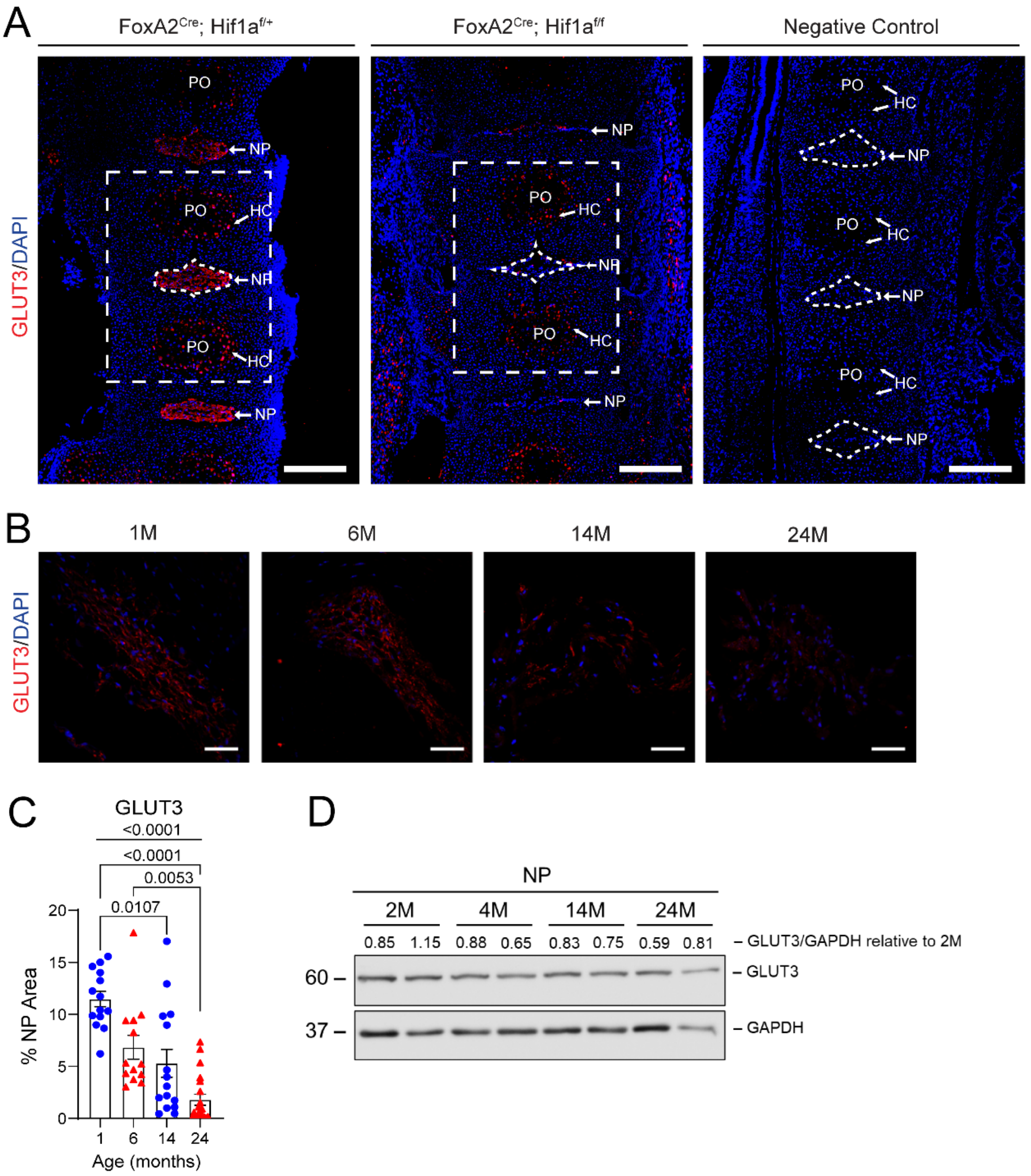

Fig. S1. Conditional knockout of Hif1a in NP cells leads to loss of GLUT3, and GLUT3 expression is lost with age. (A) Example immunohistochemistry images showing glucose transporter 3 (GLUT3) expression in embryonic day 15.5 (E15.5) nucleus pulposus (NP) of FoxA2^{Cre}; Hif1a$^{f/+}$ and FoxA2^{Cre}; Hif1a$^{f/f}$ mice with negative control. PO, Primary center of ossification; HC, Hypertrophic Chondrocytes; NP, Nucleus Pulposus. Scale Bar = 100 μm. (B, C) Representative images and quantification of GLUT3 in wildtype (BL6/J) mice with aging (n = 5 mice/ timepoint; 2-4 discs/animal, 14-20 discs/timepoint). Scale Bar = 50 μm. (D) Western blot of GLUT3 expression in wildtype (BL6/J) mice with aging, from 2-months (2M) to 24-months (24M) (n = 2 mice/timepoint; 20 discs/animal).

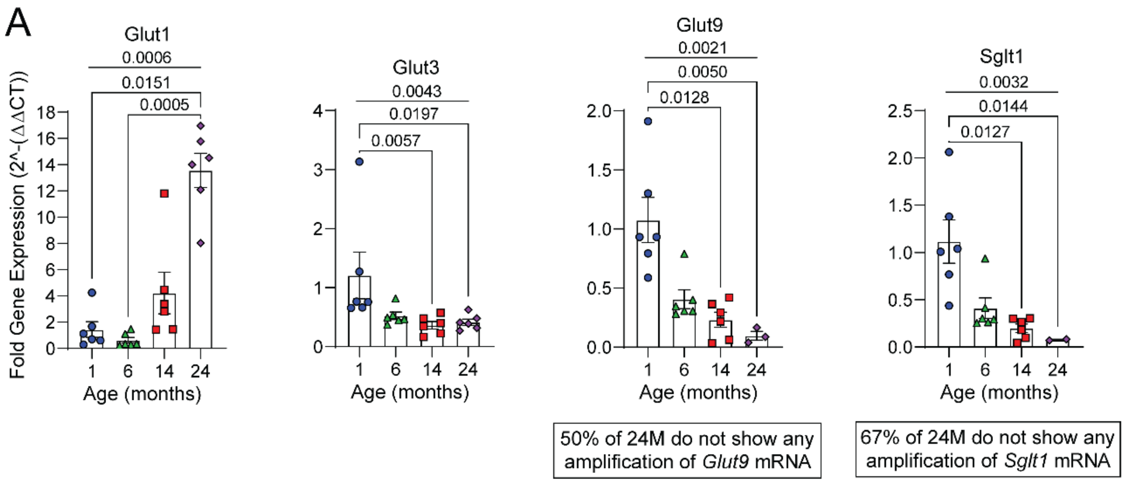

Fig. S2. mRNA expression of glucose transporters in wildtype (BL6/J) mice with aging. (A) Quantitative real time polymerase chain reaction (qRT-PCR) of glucose transporters, glucose transporter 1 (*Glut1*), glucose transporter 3 (*Glut3*), glucose transporter 9 (*Glut9*), and sodium/glucose cotransporter 1 (*Slgt1*) in 1-month (1M), 6-month (6M), 14-month (14M), and 24-month (24M) wildtype (BL6/J) mice (n = 6 mice/genotype; 20 disc/animal).

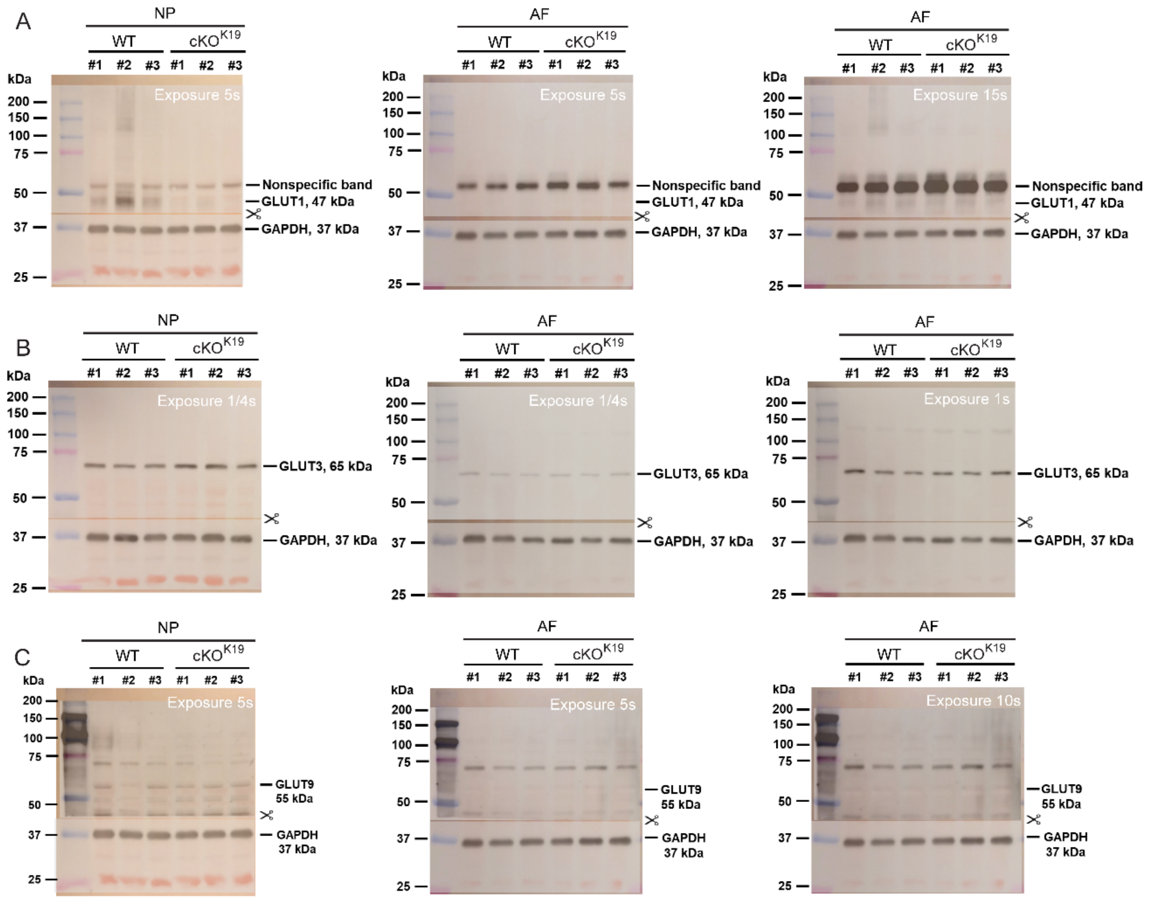

All GAPDH blots exposure time (Exp.) 1/8s
GLUT1 was probed first, stripped, and re-probed with GLUT9.
GLUT3 was run on a seperate blot.

Fig. S3. Western Blots showing GLUT1, 3 and 9 levels in NP and AF of WT and Glut1cKO^{K19}. (A) Glucose transporter 1 (GLUT1) levels in wildtype (WT, Glut1$^{f/f}$) and Keratin-19-Cre-Glut1-conditional knockout (Glut1cKO^{K19}) nucleus pulposus (NP) and annulus fibrosus (AF). (B) GLUT3 levels in Glut1 WT and Glut1cKO^{K19} NP and AF. (C) Glucose transporter 9 (GLUT9) levels in GLUT1 WT and Glut1cKO^{K19} NP and AF. The blots were first probed for GLUT1 stripped and re-probed for GLUT9. AF and NP blots are run with identical amount of tissue protein. AF blot shows identical exposure time of 5 seconds and a higher exposure time of 15 seconds to that of NP blot. All glyceraldehyde 3-phosphate dehydrogenase (GAPDH) blots had identical exposure time of 1/8 seconds. All blots are overlaid with images of Ponceau Red stained membranes to show molecular weight markers and indicates position where membranes were cut. Blots shown are for n = 3 mice/genotype; 20 discs/animal.

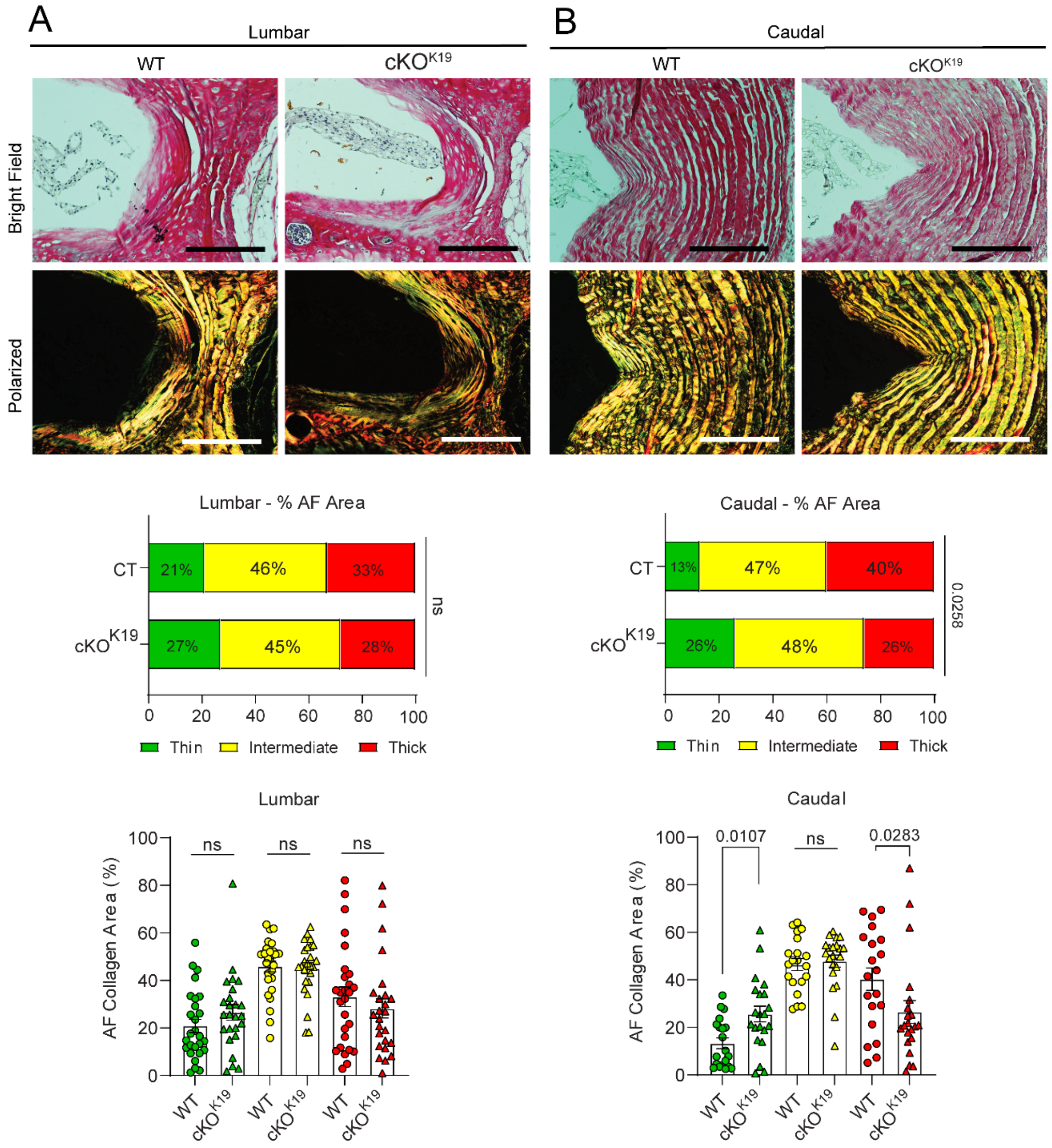

Fig. S4. Picrosirius Red staining and polarized light imaging of Glut1cKO^{K19} discs. (A, B) Representative brightfield and polarized light images of Glut1 wildtype (WT) and Keratin19^{CreERT}-Glut1-conditional knockout (Glut1cKO^{K19}) lumbar and caudal discs. And, quantification of distribution and individual abundance of thin, intermediate, and thick fibers. (n = 8 WT, 7 Glut1cKO^{K19} mice; 6 lumbar and 3 caudal discs/animal). Statistical significance in distribution was determined using a χ^2 test. Statistical significance of thin, intermediate, and thick fiber size abundance was determined using Mann-Whitney test. Quantitative measurements represent mean ± SEM.

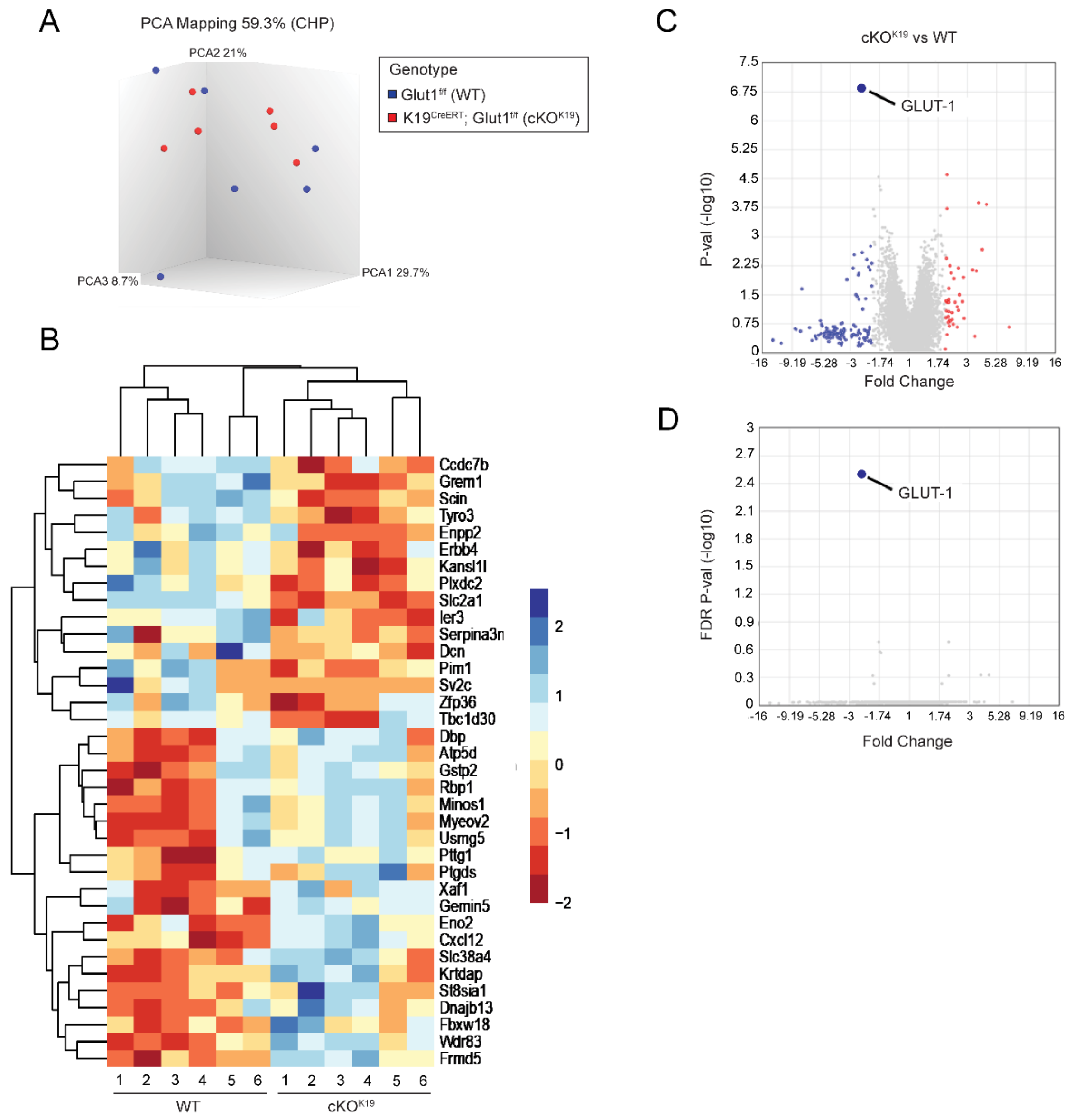

Fig. S5. Glut1cKO^{K19} mice have a similar transcriptomic profile to that of Wildtype mice. (A) Transcriptomic profiles from 6 wildtype (WT) and 6 Keratin-19-Cre-Glut1-conditional knockout (Glut1cKO^{K19}, cKO ^{K19}) mice were compared by three-dimensional principal component analysis (PCA). (B) Heatmaps of differentially expressed genes (DEGs) using Z-score between Wildtype and Glut1cKO^{K19}. (C) Volcano Plot showing the relationship between fold change ($\geq$ ±2-fold) and p-value (< 0.05), Glucose transporter 1 (Glut1/*Slc2a1*) is pointed out along with significantly upregulated (red dots) and downregulated (blue dots) DEGs. (D) Volcano Plot showing the relationship between fold change (>±2-fold) and false discovery rate (FDR) p-value (<0.05), the only gene meeting this FDR significance cutoff is Glut1/*Slc2a1*. (n = 6 mice/genotype; 20 discs/animal).

Table S1. qPCR Primers

Gene	Forward (5' to 3')	Reverse (5' to 3')
Glut1	GGCCTGACTACTGGCTTTGT	TGCATTGCCCATGATGGAGT
Glut3	GGTGGAGCGGTGAAGATCAG	GAGATGGGGTCACCTTCGTT
Glut9	GATGATGTCTGTCCTGGATGTAG	GAGTGATGTCGGGTGTCTTT
Sglt1	GGATCAGGTCATTGTGCAGC	TGGTGTGCCGCAGTATTTCT
Hprt	CGAGATGTCATGAAGGAGATGG	AGCAGGTCAGCAAAGAACTTA
